# Adults' Experiences and Perceptions of Electronic Devices for Diabetes Self‐Management: A Qualitative Evidence Synthesis

**DOI:** 10.1111/nhs.70348

**Published:** 2026-05-04

**Authors:** Vanesa Alcántara‐Porcuna, Beatriz Rodríguez‐Martin, Daniela Avello

**Affiliations:** ^1^ Department of Nursing, Physiotherapy, and Occupational Therapy, Faculty of Health Sciences University of Castilla‐La Mancha Talavera de la Reina Spain; ^2^ Technological Innovation Applied to Health Research Group (ITAS), Faculty of Health Sciences University of Castilla‐La Mancha Talavera de la Reina Spain; ^3^ ABC‐Age Research Group Cuenca Spain; ^4^ Network for Research on Chronicity, Primary Care, and Health Promotion (RICAPPS) Faculty of Health Sciences Talavera de la Reina (Toledo) Spain; ^5^ Department of Occupational Therapy, Health Sciences School, Faculty of Medicine Pontificia Universidad Católica de Chile Santiago Chile; ^6^ Centro de Desarrollo de Tecnologías de Inclusión (CEDETI‐UC) Pontificia Universidad Católica de Chile Santiago Chile

**Keywords:** adults with type 1 diabetes, qualitative evidence synthesis, self‐management, technology

## Abstract

Electronic devices are increasingly used to support self‐management in adults with type 1 diabetes (T1DM). However, understanding patient perceptions and lived experiences is essential to ensure effectiveness, equitable accessibility, and sustained acceptance. This qualitative evidence synthesis explored how adults with T1DM perceive and experience electronic devices for self‐management, focusing on benefits, barriers, and contextual factors influencing use. A systematic search across six databases was conducted using the Cochrane Qualitative and Implementation Methods Group approach. Qualitative studies published up to December 2024 were included. Data were analyzed using descriptive thematic synthesis and reported, following PRISMA and ENTREQ guidelines. Twenty‐seven studies encompassing 522 participants were included. Eight key review findings emerged: Digital devices foster empowerment, encourage positive lifestyle changes, and could improve interactions with healthcare professionals. However, sustained use and engagement were shaped by contextual and emotional factors. Significant barriers include financial constraints, technical and usability problems, and limited continuous psychological and clinical support. Social support and integration into healthcare systems also influenced engagement. Electronic devices for T1DM self‐management are complex socio‐technical interventions whose potential depends on truly person‐centered, equitable, supportive care.

**Trial Registration:** PROSPERO: CRD42024572860

## Key Points

- Electronic technologies, such as continuous glucose monitoring systems, insulin pumps and mobile health applications, may support autonomy, confidence, and perceived well‐being in adults with type 1 diabetes; however, acceptance and sustained use are shaped by everyday user experiences and contextual conditions.
- Technical demands, emotional responses (e.g., distress or anxiety), financial constraints, variable social and service‐level support can hinder adoption, particularly for people with lower digital literacy or limited access to tailored training and follow‐up.
- Integrating adults' experiences and preferences into design, education, and clinical support is essential for person‐centered, equitable implementation of technology enabled diabetes self‐management.

## 1 Introduction

Type 1 diabetes mellitus (T1DM) is an autoimmune chronic disease that affects approximately 8.5 million people worldwide (Grattoni et al. 2025). Its prevalence is growing, with estimates that in 2040, it will affect 13.5–17.4 million people (Gregory et al. 2022). The clinical management of type 1 diabetes mellitus requires an individualized therapeutic regimen characterized by glycemic monitoring and exogenous insulin administration to maintain glycemic control and prevent severe long‐term complications (Grattoni et al. 2025; Pillay et al. 2015).

With the advancement of technology, electronic devices such as continuous glucose monitoring systems (CGMs), insulin pumps (continuous subcutaneous insulin infusion (CSII)), and mobile health apps have become integral in assisting people with T1DM self‐management. These technologies offer real‐time data, significantly enhancing decision‐making and improving clinical outcomes such as quality of life, time in range, glycosylated hemoglobin values (HbA1c), and the risk of hypoglycemia (Biester et al. 2022; Pintaudi et al. 2022; Toledo‐Chavarri et al. 2024). Previous studies have demonstrated the potential benefits of these technologies in people with diabetes. For example, tablet‐based applications have been shown to enhance self‐dependence and promote positive behavior changes among older adults with type 2 diabetes mellitus (T2DM) (Alkawaldeh et al. 2020). Mobile health applications such as the Bant II app facilitate the self‐monitoring of blood glucose, diet, and physical activity in people with T2DM (Goyal et al. 2016). Despite these advancements, notable barriers remain, including technical difficulties and a lack of personalized feedback (El‐Gayar et al. 2013). Studies have also highlighted users' mixed feelings about digital health services, where increased patient involvement and empowerment come with feelings of ambivalence and uncertainty (Öberg et al. 2018). Technological devices, such as continuous glucose monitors and insulin pumps, have improved self‐management behaviors and psychological well‐being. However, studies have highlighted users' mixed feelings about digital health services, emphasizing the importance of age‐appropriate interventions and technological fluency (Phiri et al. 2022).

General perceptions of diabetes technology adoption reveal both facilitators and barriers. In this sense, a European survey of adults with T1DM identified significant benefits and challenges associated with these technologies (Penfornis et al. 2023). Patient portals have also been useful, especially among emerging adults, providing easy access to health information and facilitating communication with health care providers (Scheckel et al. 2023). Personalized interventions through health applications can also significantly enhance self‐management by addressing individual needs (Stephen et al. 2022). Community‐led surveys have revealed that real‐world experiences with diabetes technology are generally positive, although affordability and usability remain key concerns (Read et al. 2023). While there is a growing body of literature on the use of electronic devices for self‐management of diabetes, the perceptions and experiences of adults with T1DM regarding these technologies remain underexplored. There is also a lack of comprehensive reviews that synthesize qualitative evidence across different cultural and geographical contexts. Although previous quantitative reviews and mixed‐method studies have evaluated the clinical effectiveness and use of technologies among people with diabetes, the aim of this study was not to analyze the effectiveness of these devices, but rather to interpret how users experience and attribute meaning to the use of these technologies. For this reason, a qualitative evidence synthesis (QES) was considered the most appropriate methodological approach, given its capacity to explore aspects that cannot be identified through quantitative aggregation, such as contextual influences and experiential and meaning‐based dimensions.

Therefore, this qualitative evidence synthesis was conducted to understand the perceptions and experiences of adults with T1DM regarding the use of electronic devices to promote self‐management. This review answered the following questions:

How do adults with T1DM perceive and experience the use of ICTs in day‐to‐day self‐management (e.g., meanings, emotions, and perceived benefits and drawbacks)?

What factors shape acceptance, sustained use, and integration of ICTs into everyday life and routine care (including perceived barriers, facilitators, and support needs)?

## 2 Methods

Qualitative evidence synthesis (QES) was carried out following the Cochrane Qualitative and Implementation Methods Group approach (Noyes et al. 2019). The review protocol was registered in PROSPERO and reported following the PRISMA 2020 guidelines (Page et al. 2021), enhancing transparency in reporting the synthesis of the qualitative research checklist (ENTREQ) (Tong et al. 2012). GRADE‐CERQual was applied to assess confidence in the synthesized qualitative findings, considering methodology, coherence, adequacy of data, and relevance (Lewin et al. 2018). An interpretive approach was adopted to explore in depth the experiences and perceptions related to the use of technologies for self‐management in adults with T1DM.

### 2.1 Data Sources and Search Strategy

A comprehensive search was conducted in six databases (Medline (PubMed), Scopus, Web of Science, CINHAL, ProQuest, and PsycINFO) for qualitative studies in English or Spanish—due to feasibility and the language competencies of the review team—published up to December 23, 2024.

The research team defined the search strategy in collaboration with a specialized health sciences librarian. Initially, a search string was developed based on the research question and key terms. This preliminary strategy was subsequently reviewed and refined by the librarian using controlled vocabulary, including MeSH terms and other database‐specific thesauri as appropriate. Boolean operators (AND, OR, NOT) were employed to combine conceptual blocks using descriptors such as type 1 diabetes, self‐management, digital technologies (e.g., mobile devices, mHealth, eHealth), and qualitative research approaches. Truncation was applied when necessary to enhance the sensitivity and specificity of the search strategy.

The full search strategy is available in Table S1.

### 2.2 Inclusion Criteria

The following inclusion criteria were considered: (1) Qualitative studies. We included qualitative studies using any recognized qualitative design (e.g., phenomenology, grounded theory, ethnography, qualitative descriptive studies etc.) provided that data collection and analysis were clearly qualitative and findings were reported as qualitative themes/categories supported by participant data. (2) Mixed methods studies were included if qualitative analysis data were presented independently. (3) Studies that included adults with type 1 diabetes mellitus aged between 18 and 65 years. Inclusion was restricted to adults with T1DM due to maintain conceptual and clinical coherence, as self‐management demands, technology use and care pathways differ substantially between T1DM and T2DM and between adult and pediatric/adolescent populations. Including T2DM or younger age groups would have increased heterogeneity due to differing self‐management patterns and support structures, complicating synthesis and interpretation of experiences. (4) Eligible ICTs included continuous glucose monitoring system (CGMs), insulin pumps/CSII (including related diabetes device ecosystems), mobile or web‐based applications for self‐management, and patient portals/connected platforms supporting diabetes care.

The following exclusion criteria were used: (1) Studies that included hospitalized individuals, because their experiences are shaped by acute care and short‐term clinical decision‐making. (2) Studies which primary focus was disability‐related accessibility/assistive technology rather than experiences of ICTs for T1DM self‐management. (3) Unfinished studies or those that included secondary data analysis. (4) Studies conducted on pregnant women, because pregnancy involves additional clinical considerations, such as intensive monitoring protocols and specific glycemic targets, which introduce clinical heterogeneity.

### 2.3 Data Extraction and Data Synthesis

Study selection was carried out in two phases—title and abstract screening and full‐text evaluation using COVIDENCE software. Two independent reviewers (VAP and BRM) conducted the screening and selection process; disagreements were resolved through discussion, and a third reviewer (DAS) was consulted when needed.

Key data from selected studies, including study type, objectives, data collection methods, samples, and results, were extracted and recorded in a structured spreadsheet.

A descriptive thematic synthesis was carried out for the analysis following the methods of Thomas and Harden (2008). In line with the methodological spectrum described by Flemming et al. (2019), our synthesis was primarily integrative in nature, aiming to systematically organize and summarize experiential findings across studies while remaining grounded in participants' accounts. This approach was considered appropriate given our objective to synthesize patterns of perceptions, barriers, facilitators, and contextual influences, rather than to generate new theoretical constructs. An interpretive dimension emerged during the development of analytical themes, which moved beyond descriptive aggregation to provide conceptual insights relevant to practice. We used the technical assistance ATLAS.ti version 24 software in this phase. Two researchers independently (V.A.P. and B.R.M.) conducted the data analysis, extracting and coding all relevant qualitative findings from each study, including reported themes and supporting participants quotations. The process began with line‐by‐line coding of the extracted findings. Coding was conducted inductively, allowing concepts to emerge from the data rather than applying a preexisting framework. The researchers initially coded a subset of studies independently and subsequently met to compare codes, discuss discrepancies, and agree on a preliminary coding structure. Codes were then grouped into descriptive themes and subthemes and refined iteratively as new studies were incorporated. This iterative process enabled constant comparison across studies and the reorganization of categories when necessary. In cases of disagreement, a third researcher was consulted to reach consensus.

To enhance rigor, regular meetings were held within the research team to discuss theme development and challenge emerging interpretations, ensuring that themes remained grounded in the original data while capturing patterns across studies.

From themes to review findings. Themes and subthemes were generated through thematic synthesis to organize and interpret qualitative evidence. In a subsequent analytic step, these themes were translated into CERQual review findings—concise summary statements that capture the essence of each finding and can be appraised using the four CERQual components (Lewin et al. 2018). This translation process involved a degree of abstraction, as review findings may integrate evidence across multiple themes. Therefore, the relationship between themes, subthemes, and review findings was not necessarily one‐to‐one.

### 2.4 Research Team and Reflexivity

The analysis team included experienced qualitative researchers, one of whom also had T1DM, which helped in the data interpretation process. The research team included two anthropologists (VAP and BRM), a registered nurse (BRM), and two occupational therapists (V.A.P. and D.A.S.), which allowed the findings to be interpreted from an interprofessional perspective.

### 2.5 Methodological Quality Assessment

The JBI critical appraisal tool for qualitative research (JBI) was used to critically appraise the methodological validity of the studies (Lockwood et al. 2015). Two reviewers VAP and BRM independently conducted the quality assessment of the studies. Disagreements were resolved by consensus. Importantly, the assessment of the studies' methodological quality was not used as an inclusion or exclusion criterion but rather with the final aim of determining whether the selected studies provided an adequate description of the concepts they included and whether they were methodologically sound (Campbell et al. 2011; Toye et al. 2013). The review team critically discussed quality ratings during synthesis.

### 2.6 Assessment of Confidence in the Synthesis Findings

To assess the level of confidence in the synthesized findings, the GRADE‐CERQual approach (confidence in the evidence from reviews of qualitative research) was applied (Lewin et al. 2018), considering the methodological quality of the studies, coherence of the findings across studies, adequacy of the supporting data, and relevance to the review question. After each of the four elements were assessed, an evaluation of the overall confidence in the findings supporting the review's discovery was issued. CERQual assessments were adjudicated to independently by two reviewers (VAP and BRM); discrepancies were resolved through discussion and, where necessary, a third reviewer (DAS). Evidence profiles and confidence judgments were documented for each review finding.

## 3 Results

**FIGURE 1:**
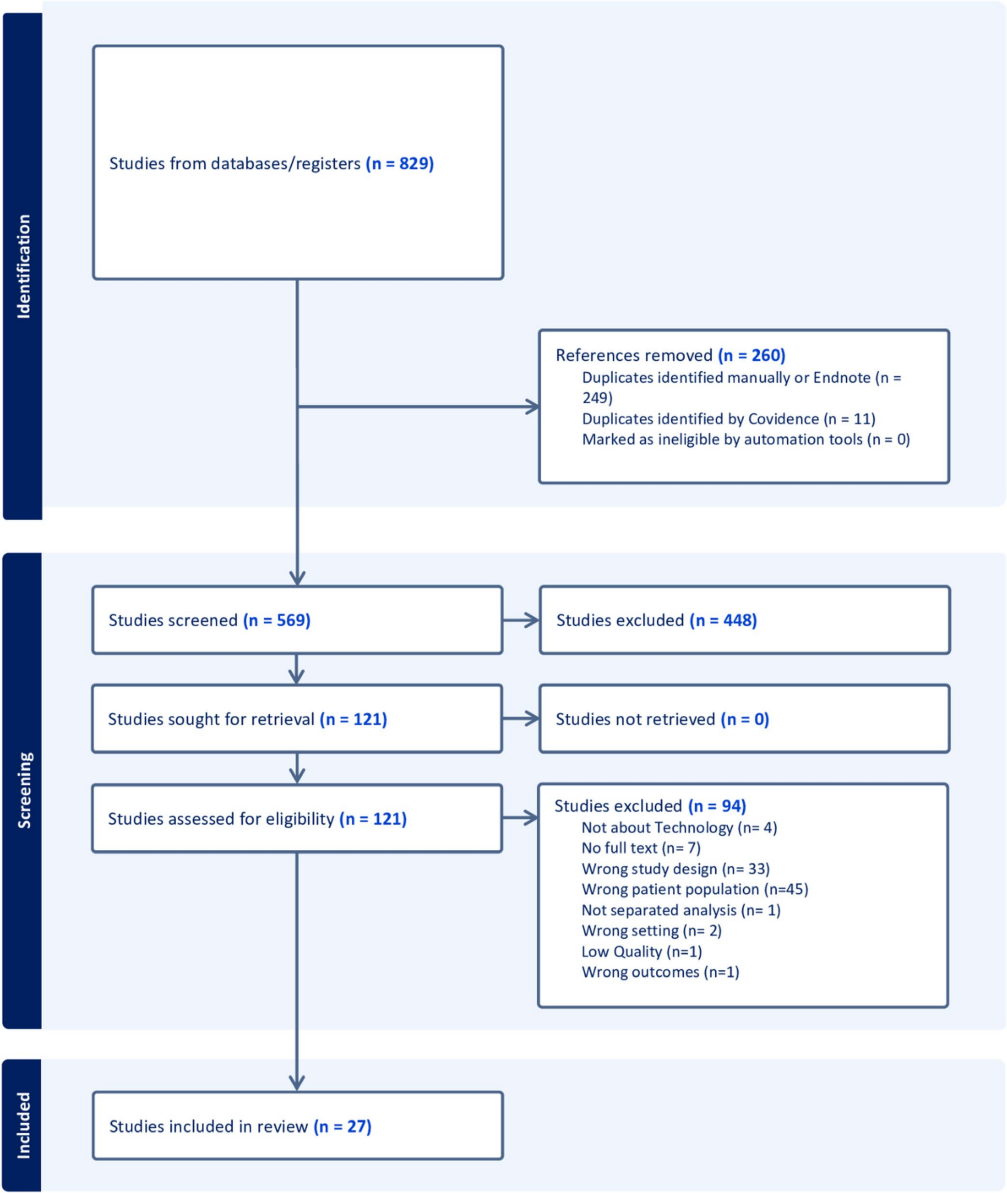
PRISMA flow diagram.

The searches retrieved 829 records and 260 duplicates were removed. Of these, 249 were removed manually or through Endnote, and 11 were removed automatically by Covidence, leaving 569 articles for the titles and abstracts screening. Subsequently, 448 studies were excluded during title and abstract screening. A total of 122 full text articles were assessed for eligibility. Of these, 94 studies were excluded for various reasons. Finally, 27 studies (Armstrong and Powell 2009; Barth et al. 2024; Clausi and Schneider 2017; Dehnavi et al. 2022; Fergie et al. 2016; Franklin et al. 2019; Griggs et al. 2020; Huygens et al. 2016; James et al. 2023; Jensen et al. 2023; Knight et al. 2016; Markowitz et al. 2024; Marsh et al. 2020; Martyn‐Nemeth et al. 2019; McFadden et al. 2024; Nettleton et al. 2022; Ng et al. 2017; Oser et al. 2019; Persson et al. 2022; Ritholz et al. 2019; Sorgard et al. 2019; Stawarz et al. 2023; Vitale et al. 2024; Vloemans et al. 2017; Waite et al. 2013; Xie et al. 2023; Zhang et al. 2018) were included, capturing the experiences and perspectives of 522 adults living with T1DM. Figure 1 shows the PRISMA flow diagram of the study selection process (see Figure 1).

### 3.1 Description of Included Studies

The main characteristics of the included studies can be found in Tables 1 and 2. Most were conducted in high‐income countries, with the United States (*n* = 7) and the United Kingdom (*n* = 7) being the most represented, followed by Canada (*n* = 3), Australia (*n* = 2), and the Netherlands (*n* = 2); other countries included Norway, China, Iran, Denmark, Sweden, and Switzerland. The data were collected primarily through semi‐structured interviews (*n* = 21) and focus groups (*n* = 6). Sampling was mostly purposive or convenience‐based, with recruitment taking place in clinical settings (*n* = 11), community environments (*n* = 14), and online platforms (*n* = 2). A wide range of digital health technologies was represented across studies, including continuous glucose monitoring systems (CGMs; *n* = 6), insulin pumps (*n* = 6), mobile or web‐based applications (*n* = 10), and patient portals (*n* = 3). These technologies were primarily used to support self‐management, although concerns about their usability, reliability, and emotional burden were frequently reported.

**TABLE 1:** Main characteristics of the analyzed studies.

Study	Country	Method of data collection	Qualitative study design	Sample or participants	Setting	Place and methods of recruitment
(Oser et al. 2019)	USA	Blog posts, comments, interviews, journals	Inductive thematic approach	*N* = 67 blog posts, *N* = 717 comments, *N* = 10 interviews (age range 19–63, 36.9 avg. 70% female)	Penn State Hershey clinical	Blogs, snowball sampling from known high‐traffic diabetes blogs
(Sorgard et al. 2019)	Norway	Semistructured interviews	Critical incident technique	*N* = 23 adults with T1DM, aged 18+ (12 females, 11 males, avg. age 42), 5 insulin pump users, 18 insulin injection users	Specialist diabetes clinics	Purposeful sampling from four outpatient diabetes clinics in eastern Norway
(Ritholz et al. 2019)	USA	Semistructured interviews	Thematic analysis	*N* = 10 (4 females, 6 males, avg. age 52, 9 non‐Hispanic, 1 Hispanic, 4 using insulin pumps, 6 using multiple daily injections)	Joslin Diabetes Center	Recruited by phone and email, interviews scheduled evenings/weekends
(Zhang et al. 2018)	China	Surveys and semistructured interviews	Mixed‐methods design	*N* = 18 (12 adults, 6 parents of young patients), age avg. 26.8, 9 insulin injection users, 3 insulin pump users	Second Xiangya Hospital	Recruitment via WeChat groups and personal networks
(Ng et al. 2017)	Australia	Semistructured interviews, self‐report surveys	Constructivist grounded theory	*N* = 13 young adults with T1DM (10 females, 3 males, avg. age 20, 8 using insulin pumps)	Community	Recruitment via social media and online diabetes support groups
(Franklin et al. 2019)	UK	Semistructured interviews	Exploratory design, thematic analysis	*N* = 8 adults with T1DM (4 females, 4 males, age range 27–57), 1 insulin pump user, 7 insulin injection users	Local urban area, Clinical Research Unit	Participants selected from CRU recruitment register, ads in local university and diabetes groups
(Knight et al. 2016)	Australia	Education session, focus group	Thematic analysis	*N* = 7 adults (5 females, 2 males, age range 18–65, all with T1DM)	DAFNE Program	Participants enrolled in DAFNE, recruited through social media and campus postings
(Huygens et al. 2016)	Netherlands	Focus group (by chronic condition)	Content analysis	*N* = 14 adults with T1DM, 9 with EPOC, 7 with cardiovascular disease, avg. age 67.1	Primary care centers	Recruited from four primary care centers by health care professionals
(Nettleton et al. 2022)	UK	In‐depth photo elicitation interviews	Phenomenological analysis	*N* = 9 participants with T1DM, age range 23–61, various demographics (5 married, 3 in relationships)	Community	Convenience sampling via social media and personal networks
(Fergie et al. 2016)	UK	Semistructured interviews	Grounded theory, thematic analysis	*N* = 20 young adults with diabetes or CMHD, 10 males, 10 females (age 18–30)	Community	Recruited through higher education institutions, online forums, and guardian assistance
(Clausi and Schneider 2017)	Canada	Semistructured interviews (in person or Skype)	Phenomenology	*N* = 7 women (aged 18–22) diagnosed with T1DM (time since diagnosis 1–14 years)	Community (University)	Recruited from a university campus in Ontario via social media and criterion sampling
(Griggs et al. 2020)	USA	Semistructured interviews	Descriptive qualitative approach	*N* = 30 young adults with T1DM (66.7% females, avg. age 22.1)	Yale‐New Haven Health System	Recruited from Yale Health System, participants aged 18–30 with T1DM for at least 6 months
(Martyn‐Nemeth et al. 2019)	USA	Focus groups	Qualitative approach	*N* = 30 adults with T1DM (63% females, avg. age 30, 70% white, 20% Hispanic)	Community	Recruited through flyers at medical centers, online support groups, and previous study participants
(Marsh et al. 2020)	USA	Semistructured interviews	Qualitative approach	*N* = 27 young adults (19 females, 8 males, aged 18–26)	Community	Recruited via snowball sampling from a youth diabetes camp in San Diego
(Vloemans et al. 2017)	Netherlands	Semistructured interviews	Exploratory approach, thematic analysis	*N* = 23 adults (avg age 47.7), mixed insulin treatments	Community	Recruited from the IN‐CONTROL trial investigating CGM effects on glycemic control
(Armstrong and Powell 2009)	UK	Focus group	Qualitative approach	*N* = 12 adults using insulin pumps	National Health System	Recruited from a local diabetes clinic, three focus groups were conducted
(Waite et al. 2013)	UK	Semistructured interviews	Mixed methods (grounded theory)	*N* = 8 people with T1DM (adults, youths, children; 4 adults, 4 aged 8–16)	Community	Recruited from local diabetes support networks via intentional sampling
(Markowitz et al. 2024)	Canada	Focus group	Thematic analysis	*N* = 24 adults with T1DM (15 females, 9 males, aged 18–65)	Community	Recruited from St. Michael's Hospital via convenience sampling
(Xie et al. 2023)	Canada	Online surveys, semistructured	Phenomenological design	*N* = 16 interviews of 207 adults with T1DM (ages 18+)	Online, Quebec	T1DM registry (email invitations), inclusion criteria
(Jensen et al. 2023)	Denmark	Semistructured interviews	Interpretive descriptive	*N* = 36 adults with T1DM	Outpatient clinic, Aarhus University	Patients from prior RCT using Diabetes Flex
(Stawarz et al. 2023)	UK	Interviews, codesign	Reflexive thematic analysis	*N* = 15 adults with T1DM (ages 24–69)	Workshops and online participation	Social media, word‐of‐mouth, public posters
(Dehnavi et al. 2022)	Iran	Semistructured interviews	Framework analysis	*N* = 18; 10 physicians, 8 patients with diabetes	Endocrinology and metabolism clinics	Convenience sampling in clinics
(James et al. 2023)	UK	Semistructured interviews	Thematic analysis using the sensemaking framework	*N* = 24 young adults with T1DM (ages 18–25)	University settings across the UK	Social media, university diabetes groups, Juvenile Diabetes Research Foundation (JDRF) advertisements
(Persson et al. 2022)	Sweden	Focus group	Qualitative content analysis	*N* = 37 adults with T1DM and suboptimal glycemic control despite CSII treatment	Diabetes centers in Östergötland	Invitation letters sent to 108 eligible patients; final sample formed from those who accepted
(McFadden et al. 2024)	USA	Semistructured interviews	Interpretive phenomenological analysis	*N* = 31 college students with T1DM (ages 18–23)	Large public universities in the Southeastern USA	University disability offices, NIH Research Match, Juvenile Diabetes Research Foundation
(Barth et al. 2024)	Switzerland	Semistructured interviews	Reflexive thematic analysis	*N* = 15 adults with T1DM	Community	Endocrinology clinics, online recruitment via Prolific
(Vitale et al. 2024)	USA	Online surveys, semistructured interviews	Directed qualitative content analysis	*N* = 24 adults with T1DM (ages 18–25)	Pediatric endocrinology clinics	Email invitations from diabetes clinics, purposive sampling for diversity

Abbreviations: BMI, body mass index; CGM, continuous glucose monitor; CMHD, common mental health disorders; CSII, continuous subcutaneous insulin infusion; CSII, continuous subcutaneous insulin infusion; DAFNE, dose adjustment for normal eating; DOC, diabetes online community; EPOC, chronic obstructive pulmonary disease; Hba1c, hemoglobin A1c; *M*, mean; SD, standard deviation; T1DM, type 1 diabetes mellitus.

**TABLE 2:** Summary of findings.

Study	Aims	Intervention/electronic device	Main results
(Oser et al. 2019)	Expand the understanding of barriers and facilitators to exercise for adults with T1DM, exploring social media, blogs, and interviews.	Online blogs written by adults with T1DM	Diabetes technology and supplies can be burdensome, but family assistance can help reduce this burden. Online peer support motivates exercise. Monitoring blood sugar levels during exercise is based more on personal experience than on health care guidelines.
(Sorgard et al. 2019)	Describe the positive and negative experiences of adults with T1DM using CGM and how they cope with these situations.	Continuous glucose monitoring (CGM)	CGM provides convenient access to glucose levels, improving glycemic control during exercise and daily activities. Trend indicators and alarms help prevent hypoglycemia, and most participants found CGM accurate and reliable.
(Ritholz et al. 2019)	Explore experiences using the Sugar Sleuth mobile app integrated with Freestyle glucose monitors for glycemic variability feedback.	Sugar Sleuth, a glucose sensor‐based mobile app	Participants considered Sugar Sleuth empowering for self‐management, providing information to promote preventive actions. It alleviated worry, reduced uncertainty, and improved discussions with health care providers.
(Zhang et al. 2018)	Explore user perspectives on current diabetes apps and collaborate on developing a new app for T1DM management.	Design a new T1DM management mobile app	Identified themes for app design included improving diabetes self‐management, patient–doctor communication, and diabetes education. The development of a multifunctional app was informed by these needs.
(Ng et al. 2017)	Identify the health and well‐being needs of young adults (18–35 years) with T1DM to develop appropriate self‐management solutions.	Web‐based resources for T1DM management	Participants used online search engines and social networks for diabetes‐related information when health professionals were unavailable. Web‐based information was more convenient and cost‐effective compared to in‐person appointments.
(Franklin et al. 2019)	Explore how mobile technology can support self‐management for adults with T1DM and inform the design of a mobile app for T1DM.	Use of mobile technology	Mobile technology helps adults with T1DM make decisions, saves time, and facilitates data sharing with health care professionals. Four key visual features were identified to aid decision‐making and improve user experience.
(Knight et al. 2016)	Obtain user feedback on the usability of a mobile bolus calculator app for adults with T1DM.	Rapidcalc mobile phone app	Three main themes emerged: confidence in bolus calculations, satisfaction with diabetes diary features, and improved efficiency in insulin administration. Recommendations were made to improve usability.
(Huygens et al. 2016)	Investigate user needs for self‐management support and attitudes toward eHealth for managing chronic illness.	eHealth apps	Patients expressed a need for self‐control and showed willingness to use eHealth tools for self‐management. Concerns about anxiety, uncertainty, and health care budget cuts were noted, highlighting the importance of tailoring eHealth to patient needs.
(Nettleton et al. 2022)	Use photoelicitation to gain insights into the coping experiences of adults with T1DM.	Photo‐elicitation about technology	Technology and peer support were crucial for coping. Participants used various resources to manage the burden of diabetes, including reminders, support from health care services, and interpersonal relationships.
(Fergie et al. 2016)	Explore young adults' experiences with health‐related user‐generated content on social media in managing their health.	Social media sites	Social media provides valuable support for diabetes and mental health, offering authentic community‐building content. However, concerns about misinformation and privacy affect engagement.
(Clausi and Schneider 2017)	Explore the lived experiences of young women with T1DM and how their illness influences their self‐perception.	Self‐management/insulin pump	Three themes emerged: feeling restricted by the insulin pump, managing diabetes as part of their routine, and desiring normality without the illness.
(Griggs et al. 2020)	Explore barriers and facilitators for sleep among young adults with T1DM.	Technology gadgets	Electronic devices helped some participants relax before bed but were a barrier to sleep for others.
(Martyn‐Nemeth et al. 2019)	Investigate the challenges of hypoglycemia and how fear of hypoglycemia (FOH) affects diabetes management.	Insulin pump/other technologies	Participants experienced both benefits and burdens from insulin pumps and CGMs. Some felt overwhelmed, while others appreciated the sense of freedom when using technology less.
(Marsh et al. 2020)	Explore barriers and successes in using the EA patient portal to improve access to chronic disease self‐management.	Patient portal for chronic conditions	Key themes included obtaining health information from various sources, limited use of the portal, and desires for a more comprehensive portal to improve transition from pediatric to adult care.
(Vloemans et al. 2017)	Investigate the benefits and challenges of CGM in adults with T1DM.	Continuous glucose monitoring (CGM)	CGM provided insights into glucose variability and reduced distress for participants. However, some found CGM intrusive or frustrating due to technical issues.
(Armstrong and Powell 2009)	Explore perspectives on the Virtual Clinic for T1DM care.	Virtual Clinic for National Health System patients	Participants emphasized the importance of peer support, caution in using new technologies, and valuing experiential knowledge in diabetes care.
(Waite et al. 2013)	Identify usability issues of current apps for diabetes care, focusing on motivation and features desired by users.	Diabetes Diary mobile app	Children and youth found diabetes apps useful, especially for visualizing glucose data. This feature was considered a significant advantage for self‐management.
(Markowitz et al. 2024)	Explore meaningful outcomes beyond HbA1c in T1DM management; assess virtual care needs.	Virtual care, insulin pumps, CGM	Importance of personalized outcomes, peer support, adaptive management, positive attitudes toward virtual care with concerns about reduced interpersonal interaction
(Xie et al. 2023)	Evaluate user satisfaction and impact on hypoglycemia management and self‐efficacy.	Self‐guided web app “Support” for T1DM education	High satisfaction, decreased hypoglycemia frequency and fear, increased confidence in diabetes management.
(Jensen et al. 2023)	Explore patient experiences with flexible PRO‐based telehealth follow‐up for T1DM.	Diabetes Flex Care (PRO‐based telehealth system)	Enhanced patient reflection, increased self‐management, improved patient‐provider communication, and flexible care adaptation.
(Stawarz et al. 2023)	Explore how machine learning can support T1DM decision‐making in different context.	Artificial Intelligence (AI)‐based decision support, closed‐loop insulin systems, CGMs	Users rely on personal heuristics for routine situations but need AI support for unexpected scenarios; preference for contextual decision support rather than constant tracking.
(Dehnavi et al. 2022)	Identify patients “and physicians” perspectives on the use of health information technology in diabetes management.	Health information technologies for diabetes self‐management	Technology improves access to health care and self‐management but high costs and lack of training limit adoption; government support and user‐friendly design are key to successful implementation.
(James et al. 2023)	Explore challenges in T1DM self‐management during the transition to university and the role of AI in future diabetes technologies.	AI‐based decision support, closed‐loop insulin systems, CGMs	Transition disrupts self‐care routines; AI and closed‐loop systems may support adaptation, but social and behavioral factors require human‐centered solutions.
(Persson et al. 2022)	Explore self‐management strategies among people with T1DM on CSII treatment but with suboptimal glycemic control.	Continuous subcutaneous insulin infusion (CSII) therapy	Two main self‐management strategies: seeking flexibility and autonomy vs. preferring routine and stability. Patients struggled with device usability, social stigma, and adherence, highlighting the need for personalized education and support.
(McFadden et al. 2024)	Explore symptom management experiences among college students with T1DM using a theoretical framework.	CGMs, insulin pumps, mobile apps	Students relied on physiological awareness and technology to detect symptoms; symptom response was immediate for hypoglycemia but delayed for hyperglycemia; challenges included “rollercoasting” due to overeating for low blood glucose.
(Barth et al. 2024)	Explore the role of blood glucose prediction (BGP) technologies in T1DM self‐management.	MOON‐T1D app (BGP simulation, insulin and nutrition tracking)	BGP aids decision‐making but you introduce trust issues; users prefer contextualized insights; potential for reducing stress but risk of overreliance on predictions.
(Vitale et al. 2024)	Assess transition to self‐management, explore barriers and facilitators in care transfer.	CGMs, insulin pumps, online patient portals	Lower diabetes distress and higher adherence are linked to better glycemic control; support from peers, mental health resources, and supply access facilitation are key for successful transition.

Abbreviations: BMI, body mass index; CGM, continuous glucose monitor; CSII, continuous subcutaneous insulin infusion; DOC, diabetes online community; EA, electronic access (patient portal); FOH, fear of hypoglycemia; Hba1c, hemoglobin A1; T1DM, type 1 diabetes mellitus.

### 3.2 Quality Assessment

The methodological quality of the included studies was assessed via the JBI Critical Appraisal Checklist for Qualitative Research (Lockwood et al. 2015). The full results can be found in Table 3. Overall, most studies have demonstrated appropriate alignment between the stated methodology, data collection and analysis techniques, and the interpretation of findings (Clausi and Schneider 2017; Fergie et al. 2016; Franklin et al. 2019; Griggs et al. 2020; James et al. 2023; Marsh et al. 2020; Martyn‐Nemeth et al. 2019; Nettleton et al. 2022; Ng et al. 2017; Oser et al. 2019; Ritholz et al. 2019; Sorgard et al. 2019; Vitale et al. 2024; Vloemans et al. 2017; Zhang et al. 2018). However, recurring limitations were identified, particularly regarding reflexivity. Several studies did not report on the theoretical or cultural position of researchers or reflect on their potential influence on the research process (Armstrong and Powell 2009; Clausi and Schneider 2017; Fergie et al. 2016; Huygens et al. 2016; Knight et al. 2016; Marsh et al. 2020; Martyn‐Nemeth et al. 2019; Nettleton et al. 2022; Ng et al. 2017; Oser et al. 2019; Ritholz et al. 2019; Vloemans et al. 2017; Waite et al. 2013; Zhang et al. 2018). In addition, some studies provided insufficient detail in their data analysis, limiting the ability to assess the interpretability of findings (Franklin et al. 2019; Martyn‐Nemeth et al. 2019; Nettleton et al. 2022; Oser et al. 2019; Ritholz et al. 2019; Xie et al. 2023). Methodological appraisal informed interpretation of the synthesis; studies were not excluded based solely on quality ratings.

**TABLE 3:** Evaluation of methodological quality according to the JBI Critical Appraisal Checklist for Qualitative Research.

1	2	3	4	5	6	7	8	9	10	11
(Oser et al. 2019)	Yes	Yes	Yes	Yes	Yes	Unclear	No	Yes	Yes	Yes
(Sorgard et al. 2019)	Yes	Yes	Yes	Yes	Yes	Yes	Yes	Yes	Yes	Yes
(Ritholz et al. 2019)	Yes	Yes	Yes	Yes	Yes	Unclear	No	Yes	Yes	Yes
(Zhang et al. 2018)	Yes	Yes	Yes	Yes	Yes	No	No	Yes	Yes	Yes
(Ng et al. 2017)	Yes	Yes	Yes	Yes	Yes	Yes	No	Yes	Yes	Yes
(Franklin et al. 2019)	Yes	Yes	Yes	Yes	Yes	Unclear	Yes	Yes	Yes	Yes
(Knight et al. 2016)	Not applicable	Yes	Yes	Yes	Yes	No	No	Unclear	No	Yes
(Huygens et al. 2016)	Not applicable	Yes	Yes	Yes	Yes	No	Unclear	Yes	Yes	Yes
(Nettleton et al. 2022)	Yes	Yes	Yes	Yes	Yes	Unclear	Yes	Yes	Yes	Yes
(Fergie et al. 2016)	Yes	Yes	Yes	Yes	Yes	Yes	No	Yes	Yes	Yes
(Clausi and Schneider 2017)	Yes	Yes	Yes	Yes	Yes	Yes	Unclear	Yes	Yes	Yes
(Griggs et al. 2020)	Yes	Yes	Yes	Yes	Yes	Yes	No	Yes	Yes	Yes
(Martyn‐Nemeth et al. 2019)	Yes	Yes	Yes	Yes	Yes	No	No	Yes	Yes	Yes
(Marsh et al. 2020)	Yes	Yes	Yes	Yes	Yes	No	No	Yes	Yes	Yes
(Vloemans et al. 2017)	Yes	Yes	Yes	Yes	Yes	Yes	No	Yes	Yes	Yes
(Armstrong and Powell 2009)	Unclear	Yes	Yes	Yes	Yes	No	No	Yes	Yes	Yes
(Waite et al. 2013)	Unclear	Yes	Yes	No	Unclear	Yes	No	Yes	Yes	Yes
(Markowitz et al. 2024)	Yes	Yes	Yes	Yes	Unclear	Yes	Yes	Yes	Yes	Yes
(Xie et al. 2023)	Yes	Yes	Yes	Yes	No	Unclear	Yes	Yes	Yes	Yes
(Jensen et al. 2023)	Yes	Yes	Yes	Yes	Unclear	Yes	Yes	Yes	Yes	Yes
(Stawarz et al. 2023)	Yes	Yes	Yes	Yes	No	Yes	Yes	Yes	Yes	Yes
(Dehnavi et al. 2022)	Yes	Yes	Unclear	Unclear	No	Yes	Yes	Unclear	Yes	Yes
(James et al. 2023)	Yes	Yes	Yes	Yes	Yes	Yes	Yes	Yes	Yes	Yes
(Persson et al. 2022)	Yes	Yes	Yes	Yes	Unclear	Yes	Yes	Yes	Yes	Yes
(McFadden et al. 2024)	Yes	Yes	Yes	Yes	Unclear	Yes	Yes	Yes	Yes	Yes
(Barth et al. 2024)	Yes	Yes	Yes	Yes	No	Yes	Yes	Yes	Yes	Yes
(Vitale et al. 2024)	Yes	Yes	Yes	Yes	Yes	Yes	Yes	Yes	Yes	Yes

*Note:* 1: Study; 2: Is there congruity between the stated philosophical perspective and the research methodology? 3: Is there congruity between the research methodology and the research question or objectives?; 4: Is there congruity between the research methodology and the methods used to collect data?; 5: Is there congruity between the research methodology and the representation and analysis of data?; 6: Is there congruity between the research methodology and the interpretation of results?; 7: Is there a statement locating the researcher culturally or theoretically?; 8: Is the influence of the researcher on the research, and vice versa, addressed?; 9: Are participants and their voices adequately represented?; 10: Is the research ethical according to current criteria or, for recent studies, and is there evidence of ethical approval by an appropriate body?; 11: do the conclusions drawn in the research report flow from the analysis, or interpretation, of the data?

### 3.3 Thematic Synthesis and Development of Review Findings

**FIGURE 2:**
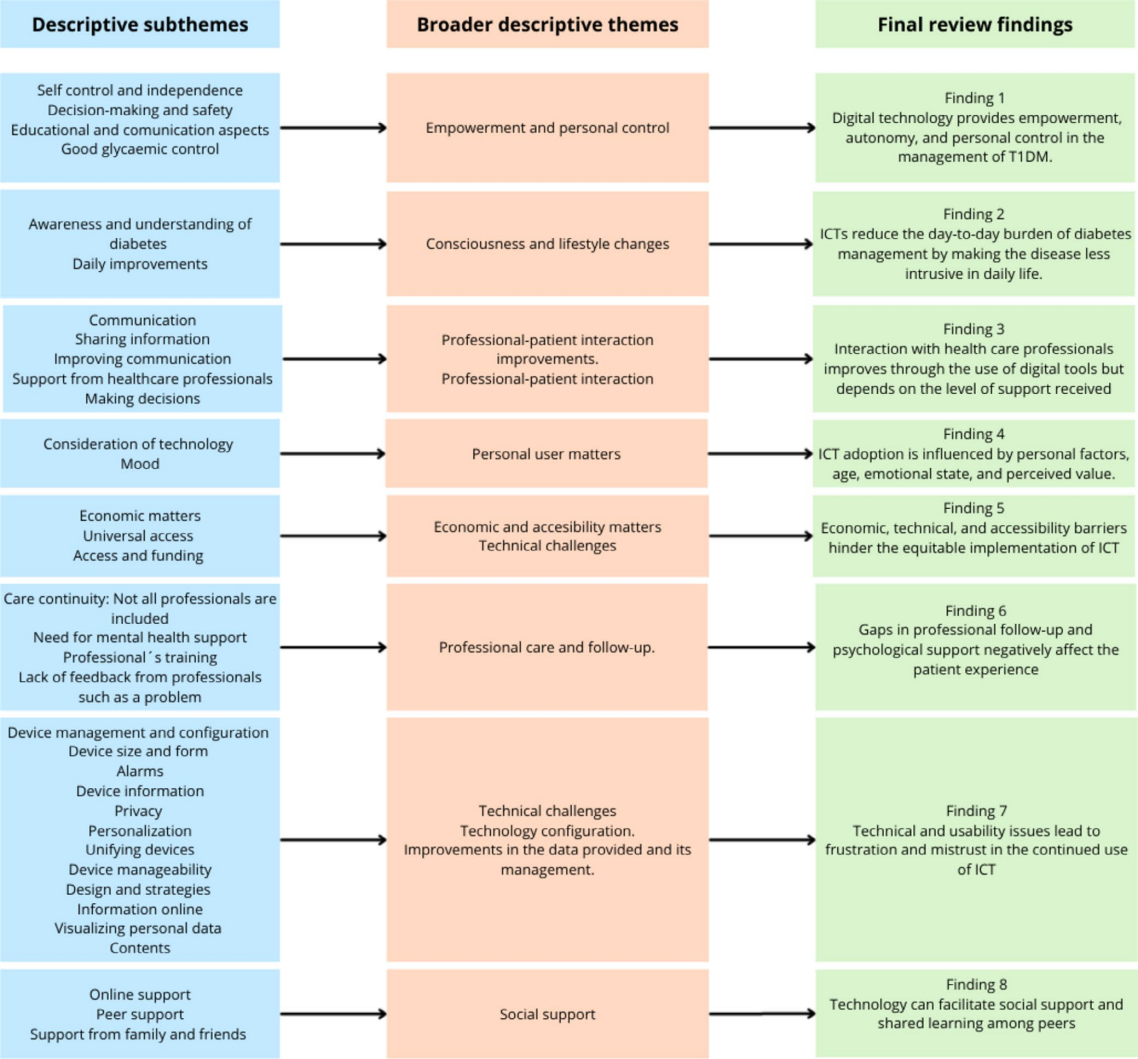
Development of final review findings from descriptive subthemes and broader descriptive themes.

The thematic synthesis generated 11 descriptive subthemes, which were subsequently grouped into broader descriptives themes through an iterative process of comparison and interpretation across the included studies The analytic structure of the synthesis is presented in the theme tree (see Figure 2) and its traceability is documented in Tables S2–S12. Building on these descriptive categories, the review team developed eight overarching review findings that capture recurrent patterns in adults´ experiences and perceptions of electronic devices for diabetes self‐management. These review findings are not only the perceived benefits of digital technologies in everyday self‐management, but also the contextual, relational, and structural factors that shaped their use. According to the studies included, participants described how electronic devices could support and share learning. At the same time, participants also identified important challenges related to technology adoption, inequitable access, insufficient professional and psychological support, and technical and usability problems. The eight review findings are presented below.

#### 3.3.1 Finding 1: Digital Technology Provides Empowerment, Autonomy, and Personal Control in the Management of T1DM

This finding was supported by 25 of the 27 studies included and reflected experiences across diverse geographical settings and technological modalities, including continuous glucose monitoring (CGM), insulin pumps, mobile applications, patients' portals, and web‐based platforms. Overall, the use of these digital technologies has been associated with an increased sense of personal control and autonomy in the routine management of T1DM. In addition, the real‐time visualization of glucose levels enabled participants to gain a deeper understanding of how factors such as diet, physical activity, sleep, and stress influence glycemic variations, thereby promoting more informed and personalized decision‐making. This increased sense of control also encompassed emotional dimensions that went beyond technical monitoring. Devices such as insulin pumps and CGM systems were mentioned as tools that enhance autonomy and reduce dependence on healthcare professionals or family members, especially in high‐risk situations such as nocturnal hypoglycemia.

Well just seeing where my blood sugars were going, and being able to keep track of everything in one location, what I was eating, my activity level, um, my insulin dosages, and then being able to see snapshots of where you went low when you went for a 2 mile walk and just compare it to a day where I sat at my desk all day…. It really helped me to understand how to better adjust my insulin dosages, to better reflect, or to have better control and fewer fluctuations. (Ritholz et al. 2019, 4)

Many participants felt that this was reflected in greater emotional well‐being, increased satisfaction with treatment, and renewed confidence following episodes of diabetes‐related fatigue. Likewise, the possibility of recording and analyzing glycemic data in real time, receiving personalized suggestions, or accessing digital health portals where health results could be shared further strengthened study participants' perception of being informed, thereby facilitating active involvement in their medical care. As one participant commented:

Mobile applications and other interactive platforms were also considered important educational tools for fostering active participation in personal care and diabetes self‐management. However, although empowerment emerged as a predominant pattern, some participants pointed out that the sense of control and the related benefits also depended on individual factors, such as frequency of use and emotional state. This means that the empowerment offered by technology is not automatic, but rather contextual and dynamic.

#### 3.3.2 Finding 2: ICTs Reduce the Day‐To‐Day Burden of Diabetes Management by Making the Disease Less Intrusive in Daily Life

This finding was supported by 23 of the 27 studies. Taken together, these studies showed that ICTs reduce the day‐to‐day burden of diabetes management by making the condition less intrusive in daily life.

Unlike Finding 1, which focused mainly on empowerment and control over personal healthcare, this finding suggests that the value of ICTs also lies in their capacity to transform the daily experience of living with T1DM. In this sense, their contribution is not limited solely to reinforcing individual control over the condition but also involves reshaping the way it is experienced and incorporated into everyday life. The studies reviewed suggest that the main benefit of these technologies is not only the improvement of glucose monitoring but also the reduction of the invasive, constant, and absorbing nature of self‐care. Tools such as continuous glucose monitoring and insulin pumps not only facilitated specific management tasks but also helped diabetes interfere less with daily routines. According to the studies reviewed, immediate access to glucose information and the ability to anticipate changes reduced the need for continuous vigilance. This made it easier to integrate treatment into activities such as work, exercise, sleep, or social participation. From this point of view, the benefit of ICTs was perceived not only in terms of efficiency or convenience but also as a reduction in the burden of living with T1DM.

(CGM) is as helpful to me on a day‐to‐day level as a wheelchair would be if I lost a leg. I mean it's hard to describe how useful it is as a technology and how valuable it is to me in terms of enabling me to live my life. (Nettleton et al. 2022, 3)

In this way, ICTs acted as a facilitator in coping with the condition and enabling a daily life that was less limited by diabetes, thanks to the reduction of interruptions, constant worries, and part of the effort required for self‐care. The studies reviewed suggested that the value of ICTs goes beyond clinical control, since they allow the disease to play a less dominant role in daily life and, in doing so, relieve part of the emotional burden associated with its management.

#### 3.3.3 Finding 3: Interaction With Health Care Professionals Improves Through the Use of Digital Tools but Depends on the Level of Support Received

The finding was supported by 13 of the 27 included studies. These studies indicated that digital tools not only facilitated the exchange of clinical information but also reshaped the way people with T1DM related to healthcare professionals. This was partly due to the perceived possibility of visualizing glycemic patterns, downloading reports, and sharing data asynchronously or remotely, which made consultations more focused, more specific, and, in many cases, more useful for adjusting treatment. Thanks to ICTs, consultations and interactions with healthcare professionals improved by shifting from a simple clinical exchange based on the one‐time review of isolated values to a more continuous, contextualized, and informed conversation about the participants' everyday experience of managing T1DM.

Overall, I really like it. For some things it is definitely easier to go in person‐like if it is an issue that takes a lot of words to describe or need to show them something. But usually mine is the short one question type, so this [messaging/emailing] has made it a lot easier to get a hold of my doctors. (Marsh et al. 2020, 4)

The included studies showed that ICTs can promote a greater sense of control and continuity of care thanks to some of their functions, such as messaging, clinical portals, and teleconsultations, which made communication more agile and accessible, especially when addressing very specific questions or making small treatment adjustments that did not require an in‐person visit. This was particularly useful for people who had just been diagnosed or during specific moments when immediate guidance or care was needed.

Even so, the findings also reflected that these benefits were not due solely to the availability of the technology; rather, their impact appeared to depend on the level of professional engagement, responsiveness, and digital competence of healthcare professionals. Study participants perceived care as more personalized when professionals were able to interpret the data generated by the devices effectively and were willing to incorporate them into the care provided. However, when support was limited or insufficient, or when professionals showed little interest in or low familiarity with the devices, the potential of ICTs was reduced. Even so, the studies reviewed suggested that digital tools had the capacity to strengthen the therapeutic relationship, although their value depended on how they were incorporated within specific professional contexts.

#### 3.3.4 Finding 4: ICT Adoption Is Influenced by Personal Factors, Age, Emotional State, and Perceived Value

This finding was supported by 17 of the 27 included studies, which suggest that the adoption and continued use of ICTs in the management of T1DM do not depend only on the technical features of the tool, but also on how each person perceives it, understands it, and integrates it into daily life. In this sense, the value that users themselves assign to these technologies seems to play an important role. When they are perceived as useful, relevant, and suited to the person's needs, their uptake appears more likely. In other hand, when they are experienced as complex, unnecessary, or even overwhelming, their use tends to become more difficult.

Age appeared in some studies as a factor worth considering, although not as sufficient explanation on its own. In several cases, younger people seemed to handle apps, digital records, and data visualization more easily, whereas some older adults described greater difficulty when interacting with these tools, especially when they had less prior experience with digital services. Even so, the findings also suggest that these differences cannot be understood only in terms of chronological age. Personal interest in technology, confidence in using it, and certain individual characteristics also seemed to influence better acceptance, including among older adults.

I should imagine someone who's really technical‐minded then I think they could have a lot of fun, you know tracking patterns and trends and working out algorithms and things like that but for someone who is in their 50s or 60s who has just developed type 2 and then eventually is put on insulin and has to record glucose levels, I don't know, I don't know. (Waite et al. 2013, 47)

In addition, the emotional dimension emerged as a relevant aspect of the relationship with ICTs. The studies suggest that the use of these tools is closely linked to the personal stage everyone is going through in relation to their condition. More positive attitudes, a sense of moving forward, and the possibility of recognizing achievements in self‐care seemed to support continued use. By contrast, emotional fatigue or a sense of overload could make this more difficult. This suggests that technology adoption should not be understood as a fixed decision, but rather as changing process shaped by the lived experience of managing T1DM in everyday life.

Taken together, the studies suggest that the incorporation of ICTs into self‐care is shaped by the interaction of personal, generational, and emotional factors. Rather than corresponding to a single user profile, these technologies seem to be accepted or rejected depending on the meaning they take on within each person's life trajectory and everyday experience of living with T1DM.

#### 3.3.5 Finding 5: Economic, Technical, and Accessibility Barriers Hinder the Equitable Implementation of ICT

Evidence for this finding was more limited and came from 4 of the 27 included studies. These studies suggest that the potential benefits of ICTs in the management of T1DM are not equally within reach for everyone. Although some participants had access to healthcare systems that provided free or subsidized medication, supplies, and certain devices, the availability of more advanced technologies was not the same in all cases. In this sense, the studies suggest that the uptake of ICTs is shaped by structural inequalities that influence who can access these tools and to what extent they can benefit from them.

Cost emerged as an important barrier to accessing and sustaining the use of technologies such as continuous glucose monitoring systems, insulin pumps, and other digital resources linked to self‐care. This was compounded by expenses related to repairs, replacements, faulty sensors, or additional features that were not always covered by funding systems. Taken together, this suggests that the adoption of ICTs does not depend only on whether they are perceived as useful, but also on whether people are realistically able to sustain their use economically and materially over time.

Honestly reordering and … juggling supplies … getting my Dexcom sensors … trying to order my pump supplies and … insulin … I have to get them from different places and somehow it never works out with my insurance. I'm always like missing something which is kind of frustrating. (Vitale et al. 2024, 8)

Alongside economic barriers, the studies also pointed to limitations related to the physical and social accessibility of devices. Some participants described certain technologies as uncomfortable to wear, inconvenient to carry, or too visible, which affected their acceptability in everyday life. In some cases, these difficulties were also linked to discomfort in social settings or to feelings of stigmatization, especially when the device made the condition more visible in public spaces or in day‐to‐day interactions.

The included studies suggest that equitable implementation of ICTs cannot be understood only in terms of availability. Their actual use also depends on economic, material, and social conditions that make it possible not only to access devices but also to incorporate them into daily life in a sustainable way. From this perspective, ICTs are not only clinical tools but also resources whose distribution and maintenance are shaped by issues of equality.

#### 3.3.6 Finding 6: Gaps in Professional Follow‐Up and Psychological Support Negatively Affect the Patient Experience

This finding was supported by 11 of the 27 included studies, which suggest that, although ICTs can support the monitoring of T1DM, their usefulness becomes limited when they are not accompanied by ongoing professional support and care that also takes patients' emotional needs into account. From this perspective, technology does not seem to replace comprehensive care but rather depends on it for its potential benefits to be fully realized.

Several studies pointed to difficulties related to continuity of care and coordination between professionals. In some cases, digital tools did not allow smooth connection with all the professionals involved in care, and certain portals included only specialist information, which contributed to a more fragmented experience of care. This suggests that, when digital systems are not well integrated, they may maintain discontinuities that already exist within healthcare.

The findings also draw attention to an important gap in psychological support. Many applications and digital platforms appeared to focus almost exclusively on glycemic control, diabetes education, and biomedical monitoring, while emotional distress and the mental health needs associated with living with T1DM remained in the background. This is particularly relevant in a condition that requires constant vigilance and can lead to fatigue, frustration, and a sense of overload.

In addition, several studies showed that limited training among some professionals in the use of digital tools, together with interactions perceived as lacking empathy or judgmental, could worsen the care experience. When patients felt poorly understood, questioned, or insufficiently supported, their trust in both professionals and the practical usefulness of ICTs tended to decrease.

I have received negative opinions and comments from health professionals who have not bothered understanding why I have not controlled my diabetes. They have just judged me and made me feel like I'm failing with my diabetes. (Ng et al. 2017, 5)

#### 3.3.7 Finding 7: Technical and Usability Issues Lead to Frustration and Mistrust in the Continued Use of ICT

This finding was supported by 23 of the 27 included studies. Taken together, these studies showed that technical and usability issues lead to frustration and mistrust in the continued use of ICTs.

The continued use of ICTs in the management of T1DM does not seem to depend only on whether they are available or on the clinical functions they offer, but also on the level of trust they inspire in those who use them and on how well they fit into everyday life. In this sense, technical problems and usability difficulties did not appear as minor inconveniences but rather as issues that could clearly affect motivation to keep using these tools.

In several studies, participants described problems with data readings, synchronization failures, loss of information, and limitations when recording values. All of this caused frustration and, in some cases, led people to return to more traditional methods of monitoring. These findings suggest that, when technology is no longer perceived as reliable, it not only loses practical usefulness but may also affect the sense of security with which people make decisions about their care. From this perspective, trust appears to be an important element in whether these tools are adopted and sustained over time. Usability was also closely linked to the physical and social experience of wearing and using devices. Some participants reported that certain devices felt uncomfortable, intrusive, or difficult to wear during exercise, rest, or intimate situations. Similarly, alarms and notifications could be experienced as constant interruptions and could generate tiredness, embarrassment, or discomfort. This suggests that acceptance of ICTs does not depend only on their accuracy but also on the extent to which they can adapt to the rhythms, spaces, and relationships that shape daily life.

And then calibration, and calibration. After three hours, another calibration. At the end I wore it for so long, but no readings just calibration. And I feel just irritated, and I think I am much better off just checking my blood sugar with a regular finger prick. (Sorgard et al. 2019, 3324)

At the same time, the studies also show that trust in and engagement with these technologies tended to increase when platforms offered intuitive interfaces, good integration between devices, automatic data logging, clear visualizations of patterns, and simple ways of sharing information with healthcare professionals. The studies suggest that sustained use of ICTs rests on a delicate balance between what they offer and the burden they create, the more reliable, understandable, and easy to integrate these tools are, the greater their potential to become a genuine source of support in the everyday management of T1DM.

#### 3.3.8 Finding 8: Technology Can Facilitate Social Support and Shared Learning Among Peers

This finding was supported by 13 of 27 included studies, which suggest that ICTs not only facilitate access to information about T1DM but also expand opportunities for social support and shared learning among people living with the condition. In this sense, digital communities, social media, and data‐sharing options seem to open up spaces where users can exchange practical advice, feel recognized by others, and find ways of coping with everyday life based on lived experience, something that does not always find a place in more formal clinical settings.

Some participants particularly valued the speed with which they could get answers, the possibility of comparing experiences with other people with T1DM, and the emotional support that came from feeling understood by those going through similar difficulties. This suggests that technology serves not only an informational role, but also a relational one, by helping to reduce isolation and giving value to everyday forms of knowledge that often remain outside professional discourse.

At the same time, the findings also show that the relevance of this kind of digital support was not the same for everyone. When strong in‐person networks were already in place, such as support from family or friends, online communities tended to play a more complementary role. By contrast, for those with less support in their immediate environment, these digital spaces could take on a much more important role as a source of motivation, guidance, and day‐to‐day support.

In addition, some technologies have made it possible to extend that support beyond other people with T1DM by allowing family members, cohabitants, or other close contacts to access real‐time glucose data and provide support or step in when needed.

‘If I get very drunk and I'm passed out, he'll come up to […] see if everything is OK’; ‘not really anything to do with my diabetes but just in general just providing like that emotional support’; ‘my other half have access to it so that is brilliant’; ‘I've then downloaded the app that, um, the rapid calc […] was actually recommended to me by other people with type one diabetes through the support group’. (James et al. 2023, 8)

### 3.4 Certainty of the Evidence

The certainty of the eight findings was assessed using the GRADE‐CERQual approach (Lewin et al. 2018), which considers four components: methodological limitations, coherence, adequacy of data, and relevance of the contributing studies. Summary results are presented in Table 4, with a component‐by‐component breakdown in Supplementary Table 13.

**TABLE 4:** CERQual summary of qualitative findings.

Summary of review finding	Studies contributing to the review finding	CERQual assessment of confidence in the evidence	Explanation of CERQual assessment
Finding 1: Digital technology provides empowerment, autonomy, and personal control in the management of type 1 diabetes	(Armstrong and Powell 2009; Barth et al. 2024; Clausi and Schneider 2017; Dehnavi et al. 2022; Fergie et al. 2016; Franklin et al. 2019; Huygens et al. 2016; James et al. 2023; Jensen et al. 2023; Markowitz et al. 2024; Marsh et al. 2020; Martyn‐Nemeth et al. 2019; McFadden et al. 2024; Nettleton et al. 2022; Ng et al. 2017; Oser et al. 2019; Persson et al. 2022; Ritholz et al. 2019; Sorgard et al. 2019; Stawarz et al. 2023; Vitale et al. 2024; Vloemans et al. 2017; Waite et al. 2013; Xie et al. 2023; Zhang et al. 2018)	High confidence	This finding is supported by more than 20 studies, conducted in diverse geographical and cultural contexts, which provides high relevance and adequacy. The consistency across studies is high, with no notable contradictions, which supports coherence. Additionally, the studies demonstrate good methodological quality.
Finding 2: ICTs reduce the day‐to‐day burden of diabetes management by making the disease less intrusive in daily life	(Barth et al. 2024; Clausi and Schneider 2017; Dehnavi et al. 2022; Franklin et al. 2019; Griggs et al. 2020; Huygens et al. 2016; James et al. 2023; Jensen et al. 2023; Knight et al. 2016; Markowitz et al. 2024; Martyn‐Nemeth et al. 2019; McFadden et al. 2024; Nettleton et al. 2022; Ng et al. 2017; Oser et al. 2019; Persson et al. 2022; Ritholz et al. 2019; Sorgard et al. 2019; Stawarz et al. 2023; Vitale et al. 2024; Vloemans et al. 2017; Xie et al. 2023; Zhang et al. 2018)	High confidence	This finding is also well supported by numerous high‐quality studies, with consistent results showing improvements in health awareness, habits, and emotional well‐being. The studies come from multiple regions, which enhances their transferability (relevance and adequacy) and strengthens coherence. No significant methodological limitations were identified.
Finding 3: Interaction with health care professionals improves through digital tools but depends on the level of support received.	(Franklin et al. 2019; Huygens et al. 2016; Jensen et al. 2023; Knight et al. 2016; Markowitz et al. 2024; Marsh et al. 2020; Nettleton et al. 2022; Ng et al. 2017; Ritholz et al. 2019; Sorgard et al. 2019; Vitale et al. 2024; Waite et al. 2013; Zhang et al. 2018)	Moderate confidence	Although a clear trend toward improved professional—patient relationships is observed, the results largely depend on the institutional context and the level of professional engagement. This introduces some variability (moderate coherence). Despite the overall good quality of studies, not all explore this aspect in depth. Relevance is good but not consistent across all contexts.
Finding 4: The adoption of ICT is influenced by personal factors, age, emotional state, and perceived value.	(Barth et al. 2024; Clausi and Schneider 2017; Dehnavi et al. 2022; James et al. 2023; Jensen et al. 2023; Markowitz et al. 2024; Martyn‐Nemeth et al. 2019; McFadden et al. 2024; Nettleton et al. 2022; Persson et al. 2022; Ritholz et al. 2019; Sorgard et al. 2019; Stawarz et al. 2023; Vitale et al. 2024; Vloemans et al. 2017; Waite et al. 2013; Xie et al. 2023)	Moderate confidence	This finding is supported by methodologically sound studies, but there is clear variability in user experiences depending on age, technological skills, and emotional state, which affects coherence. Additionally, some studies do not address all factors comprehensively. Nevertheless, its relevance is high across different contexts.
Finding 5: Economic, technical, and accessibility barriers hinder the equitable implementation of ICT.	(Barth et al. 2024; Dehnavi et al. 2022; Sorgard et al. 2019; Vitale et al. 2024)	Low confidence	Few studies explicitly address this issue, and most come from specific contexts (Norway, Iran, USA, Switzerland), which limits their adequacy and global relevance. Additionally, coherence is limited, as the reported barriers vary significantly between countries. Although the evidence is valid, it is scarce and does not always explore the actual impact in depth.
Finding 6: Gaps in professional follow‐up and psychological support negatively affect the patient experience.	(Barth et al. 2024; Dehnavi et al. 2022; James et al. 2023; Jensen et al. 2023; Markowitz et al. 2024; Marsh et al. 2020; Nettleton et al. 2022; Ng et al. 2017; Vitale et al. 2024; Xie et al. 2023; Zhang et al. 2018)	Moderate confidence	There is moderate support from several studies regarding the lack of continuity in professional support, although the degree of impact varies depending on the health care system. This introduces inconsistency (moderate coherence). Relevance is good, as it spans different contexts, but some studies address this issue only indirectly.
Finding 7: Technical and usability issues lead to frustration and mistrust in the continued use of ICT.	(Armstrong and Powell 2009; Clausi and Schneider 2017; Dehnavi et al. 2022; Fergie et al. 2016; Griggs et al. 2020; James et al. 2023; Jensen et al. 2023; Knight et al. 2016; Markowitz et al. 2024; Martyn‐Nemeth et al. 2019; McFadden et al. 2024; Nettleton et al. 2022; Ng et al. 2017; Oser et al. 2019; Persson et al. 2022; Ritholz et al. 2019; Sorgard et al. 2019; Stawarz et al. 2023; Vitale et al. 2024; Vloemans et al. 2017; Waite et al. 2013; Xie et al. 2023; Zhang et al. 2018)	High confidence	This finding is widely supported by many studies conducted in various contexts. Negative experiences related to technical aspects (failures, complex interfaces, poorly managed data) are consistent and recurrent. Adequacy is strong, as the studies include participants of different ages and levels of digital literacy. Methodological quality is generally good.
Finding 8: Technology can facilitate social support and shared learning among peers.	(Armstrong and Powell 2009; Clausi and Schneider 2017; Dehnavi et al. 2022; Fergie et al. 2016; James et al. 2023; Markowitz et al. 2024; Marsh et al. 2020; Nettleton et al. 2022; Ng et al. 2017; Oser et al. 2019; Vitale et al. 2024; Xie et al. 2023; Zhang et al. 2018)	Moderate confidence	Studies consistently show that ICT facilitates sharing among people with type 1 diabetes, but they are fewer in number, and not all explore this aspect in depth. Coherence is good but limited by the amount of available data. Relevance is high, although some cultural settings may influence the applicability of the finding.

The resulting confidence profile was predominantly high or moderate. Three findings were rated as high confidence (Findings 1, 2, and 7), four as moderate confidence (Findings 3, 4, 6, and 8), and one as low confidence (Finding 5).

The three high‐confidence findings, empowerment and autonomy in T1DM self‐management (Finding 1), reduction of the daily burden of the disease (Finding 2), and technical problems generating frustration and distrust in ICT use (Finding 7), were supported by a large number of studies from diverse geographical and cultural contexts, with sufficient data, good coherence, and only minor methodological concerns.

The four moderate‐confidence findings also had relevant empirical support, though with limitations in one or more CERQual components. Findings on interaction with healthcare professionals (Finding 3), the influence of personal and emotional factors on ICT adoption (Finding 4), the lack of professional follow‐up and psychological support (Finding 6), and the role of technology in peer social support and learning (Finding 8) showed moderate coherence and data adequacy, some variability across institutional, healthcare, social or cultural contexts, and in some cases uneven exploration of the phenomenon across contributing studies. The only low‐confidence finding concerned economic, technical, and accessibility barriers to equitable implementation (Finding 5). Few studies addressed this issue explicitly; the evidence came from a limited number of countries, and the barriers described varied considerably across them, resulting in low coherence, limited data, and moderate relevance, alongside some methodological concerns.

Taking together, these CERQual ratings make explicit the degree of confidence that can be placed in each finding and provide a more transparent basis for their interpretation in relation to clinical practice, health policy, and future research.

## 4 Discussion

This QES suggests that in adults with T1DM, ICTs should not be understood solely as tools for monitoring glucose levels or adjusting treatment. Rather, they emerged that reshape how diabetes is experienced and managed in everyday life. Although there are technical devices, such as continuous glucose monitoring systems (CGMs), insulin pumps, and mobile applications that were associated with a greater perception of control, autonomy, and capacity for self‐monitoring, their impact appeared to extend beyond strict clinical outcomes. They also influenced the emotional experience of living with the condition, relationships with healthcare professionals, and the way treatment is incorporated into daily routines. In this sense, although electronic devices are often perceived as valuable tools for self‐management in adults with T1DM, their adoption, acceptability, equity, and usability remain shaped by technical, emotional, and practical barriers.

In line with previous studies, our findings show that the user experience does not depend only on the technical effectiveness of these devices, but also on the individual's capacity to adapt, the conditions of their everyday environment, and the support they receive (Greenhalgh et al. 2017; Öberg et al. 2018; Orlikowski 2007; Toledo‐Chavarri et al. 2024).

One of the most relevant contributions of this QES is that it allows empowerment to be understood not as an automatic consequence of access to technology, but as a process that is situated, dynamic, and shaped in relation to each person's concrete experience. In line with previous literature, having access to real‐time information, being able to identify glycemic patterns, and receiving recommendations tailored to the individual seems to support the capacity to make informed decisions and to take a more active role in self‐care (Anderson and Funnell 2010; Penfornis et al. 2023; Phiri et al. 2022). However, our findings suggest that this empowerment does not arise simply from having more data, but from that data being understandable, interpretable, and genuinely useful in specific everyday situations. From this perspective, ICTs are valuable not only because of their potential contribution to clinical control, but also because in some cases, they may ease part of the mental burden associated with constant monitoring and make living with the condition more manageable. This extends what has already been suggested in previous studies about their potential to reduce anxiety and support autonomy (Goyal et al. 2016; Huat et al. 2019; Kime et al. 2023).

This distinction is important for understanding the ambivalence that ran through many of the included studies. While some people experienced these technologies as a way of gaining freedom, others experienced them as an additional burden, a source of frustration, or even as something that intruded on their privacy. As Barnard et al. (2017) and Toledo‐Chavarri et al. (2024) had already pointed out, the impact of ICTs cannot be assessed solely in terms of their clinical accuracy or their monitoring capacity. Our findings suggest that their meaning also depends on the practical and emotional effort involved in incorporating them into everyday life. The need for constant supervision, interruptions during personal or social activities, and the sense of overload associated with some devices may reduce satisfaction with their use and lead to more intermittent patterns of use (Sorgard et al. 2019; Toledo‐Chavarri et al. 2024). On this way, the balance between functional usefulness and emotional cost emerges as a key dimension for understanding the real value of these technologies in T1DM self‐management.

Another relevant finding is that ICTs do not seem to function only as technical tools, but also as resources that shape the care relationship. Participants particularly valued the possibility of sharing data, receiving quick responses, and maintaining more continuous communication with their healthcare team. However, this QES also suggests that this potential does not depend only on the presence of the technology itself, but on whether professionals are willing to interpret it, make sense of it, and genuinely integrate it into care. This view is consistent with previous studies showing that the clinical value of these tools is shaped both by professionals' training and by their effective incorporation into clinical practice (Reidy et al. 2018). On this way, technology does not strengthen the therapeutic relationship on its own, but it can help enable care that is more continuous, more personalized and more responsive when adequate professional support is in place.

At the same time, this QES makes clear that digital innovation, on its own, does not overcome the limitations of truly comprehensive care. Several studies repeatedly highlighted difficulties related to lack of continuity of care, fragmented follow‐up, poor coordination between professionals, and the absence of psychological support. This suggests that, although ICTs can improve monitoring and make communication more fluid, they do not automatically address the emotional and relational needs involved in living with T1DM. In this respect, our findings are consistent with a person‐centered care perspective, which recognizes that patients' emotions, values, and life context are not secondary elements, but an essential part of care (Epstein and Street 2011).

In addition, our results suggest that the adoption and continued use of these technologies are strongly shaped by subjective and contextual factors. Age, familiarity with digital environments, emotional readiness, the value each person assigns to technology, and the perception that it does or does not fit their needs all clearly influenced whether these tools were accepted or rejected. This reinforces the idea that people with T1DM do not form a homogeneous group, and that the same technology may be experienced in very different ways depending on who is using it and under what circumstances.

In this QES, trust in ICTs emerged as a key issue, but also as something easily vulnerable and closely linked to the experience. Technical failures, synchronization problems, inaccurate readings, intrusive alarms, and the physical discomfort caused by some devices were not experienced as minor inconveniences. On the other hand, these actions could weaken the trust in these tools, making it harder to use them consistently. This helps explain why technology's potential does not always lead to widespread use in everyday situations. When it does not meet people's expectations or is difficult to incorporate into daily routines, its use tends to decline over time, as previous studies have also suggested (Greenhalgh et al. 2017; Salvador‐Kelly et al. 2016). The results of this synthesis indicate that individuals place a high value on technologies characterized by intuitiveness, seamless integration with other devices, the capacity to clearly visualize patterns, automated data recording, and simplified information sharing. This underscores the necessity of user‐centered design principles and implementation strategies that are sensitive to the practical context of use.

Equity also occupied an important place in this synthesis. Despite technological advances in the management of T1DM, access to these tools remains unequal across countries, regions and social groups (Reidy et al. 2018; Toledo‐Chavarri et al. 2024). Our findings show that economic barriers are not limited to the initial cost of the device but also include maintenance expenses, repairs, replacements, and additional features.

Added to this are digital exclusion and inequalities in technological literacy, which can limit the actual use of these tools, especially among vulnerable groups, older adults, people living in rural settings, or those experiencing poverty, as the literature has also pointed out (Diana et al. 2025). From this perspective, access to ICTs should be understood not only as material availability, but as the real possibility of sustaining continued, useful, and meaningful use over time.

At the same time, the findings also show that ICTs can expand support networks beyond formal health services. Online communities, social networks, and tools for sharing data in real time enabled many people to access peer support, validate knowledge built from experience, and reduce the everyday isolation associated with the condition. This suggests that the value of ICTs lies not only in connecting the person with the healthcare system but also in enabling more distributed forms of support, learning, and security in daily life.

This synthesis suggests that the future development of ICTs for T1DM should go beyond mere technical optimization. It seems necessary to move toward solutions that are inclusive, affordable, intuitive, and sensitive both to the emotional burden and to the everyday realities of those living with the condition. From nursing practice, this implies strengthening digital education, the contextualized interpretation of data, emotional support, and continuity of care. Nurses can play an essential role not only in teaching how to use devices but also in helping ensure that they are incorporated into the lives of people with T1DM in a meaningful, feasible, and sustainable way.

Along the same lines, future research should focus not only on whether ICTs are effective, but also on the conditions that make it possible for them to become acceptable, useful, and equitable in daily life. Co‐design approaches involving people with T1DM seem especially promising, as they make it possible to develop technologies that are better aligned with their values, needs, and real‐life challenges. This approach is consistent with the principles of experience‐based design, which promotes active collaboration between patients and professionals in the development of solutions that are more responsive to users' everyday lives (Bate and Robert 2006). In addition, it may help transform the culture of care toward services that are more sustainable, more equitable, and more person‐centered (Palmer et al. 2019).

### 4.1 Strengths and Limitations of This Review

This QES has several strengths. First, it followed a rigorous methodological framework recommended by the Cochrane Qualitative and Implementation Methods Group, including the use of thematic synthesis and the application of the GRADE‐CERQual approach to assess the level of confidence in the findings. The inclusion of studies from a wide range of countries provides an enriched perspective on the perceptions and experiences of people with T1DM regarding the use of technological devices for disease management and supports the relevance of the review. In addition, the involvement of a multidisciplinary research team and the inclusion of a researcher with T1DM added depth and reflexivity to the analysis.

However, this study also has several limitations. The search was limited to articles published in English or Spanish, which may have excluded relevant studies in other languages. Likewise, certain population groups, such as adults over 65, pregnant women, and people with disabilities, were not included, which may limit the applicability of our findings to these populations.

## 5 Implications for Practice

This synthesis of qualitative studies provides a deep understanding of how people with T1DM use digital technologies for self‐management of the disease, integrating a wide variety of experiences and contexts which may be useful for managing this and other chronic diseases in the digital age. Therefore, this review has important implications for practice. The findings offer valuable insights that can inform health care providers and technology developers in improving the usability and effectiveness of electronic devices for diabetes self‐management and other chronic diseases. First, electronic devices, although promising, have the capacity to reach their full potential when they are adapted to meet the emotional, technical, and social needs of people with T1DM. Therefore, it is necessary that nurses not only provide technical support but also provide emotional support to the users of these technologies. Moreover, personalization and adaptation, digital literacy and training processes, continuous support in their use by healthcare professionals, and the elimination of access barriers are considered necessary elements to achieve their effective implementation into clinical practice, which in turn can lead to increased patient engagement and better adherence to treatment. In this sense, diabetes specialist nurses and those who monitor people with chronic diseases are the key professionals to train patients and monitor the use of these tools. Therefore, it is necessary to improve the training of these professionals, provide them with time within their working days to be able to properly monitor the use of these tools, and ensure that healthcare institutions promote, through their policies, the effective integration of electronic devices into clinical practice. Second, the results (such as improvements to the data sharing system with healthcare professionals, the ability to share messages between patients and professionals or monitor chronic illness, and adjust treatment through remote consultations) provide a solid foundation to consider when redesigning electronic devices and ITC‐based interventions from an approach that is truly person‐centered in the care of people with T1DM and other chronic diseases. Finally, the results show that incorporating patient perceptions and preferences into the design and monitoring of these tools is considered by patients to be essential for improving the user experience, acceptability, and usability of these devices.

Future studies should analyze this phenomenon in other populations with T1DM, such as adolescents, older adults, and people with various chronic conditions in addition to T1DM, to find out if there are differences in their perceptions and preferences regarding the use of electronic devices to promote self‐management.

## 6 Conclusions

Electronic devices for T1DM self‐management are not merely functional clinical tools, but complex socio‐technical interventions. While these technologies offer significant potential to enhance patient autonomy, glycemic control, physical and emotional well‐being, and reduce overall disease burden in adults with T1DM, their successful integration is deeply contingent upon the user's everyday context.

Clinical effectiveness cannot be separated from the user experience; technical demands, financial constraints, and practical barriers frequently undermine sustained engagement, acceptability, and equitable access.

The findings of this study have critical implications for nursing practice, healthcare policy, and technology development. For devices to truly support T1DM self‐management, a paradigm shift toward person‐centered care is essential. Healthcare providers, particularly diabetes specialist nurses and those who monitor people with chronic diseases, must move beyond basic technical training to offer continuous, empathetic psychological support tailored to individual digital literacy and emotional needs. At the policy level, systemic efforts are required to dismantle financial barriers and ensure equitable access to these technologies and their necessary supplies. Furthermore, technology developers must prioritize experience‐based co‐design, actively incorporating the user perspective to create more intuitive, less burdensome devices.

Despite the growing body of literature, significant gaps remain that must inform subsequent investigations. Current evidence predominantly reflects the experiences of specific demographic profiles, highlighting a pressing need to investigate technological adoption among underrepresented and vulnerable populations, including older adults, individuals with lower digital literacy, and those facing socioeconomic marginalization. Additionally, exploring how these digital experiences translate to populations managing multiple chronic conditions alongside T1DM will be crucial for guiding the development of more inclusive, adaptive, and patient‐sensitive healthcare interventions in the future.

## Author Contributions

**Beatriz Rodríguez‐Martin:** conceptualization, investigation, writing – original draft, methodology, validation, writing – review and editing, formal analysis, software, supervision, project administration. **Daniela Avello:** writing – original draft, validation, writing – review and editing, supervision. **Vanesa Alcántara‐Porcuna:** conceptualization, investigation, writing – original draft, methodology, writing – review and editing, formal analysis, software, project administration.

## Funding

The authors have nothing to report.

## Ethics Statement

The authors have nothing to report.

## Conflicts of Interest

The authors declare no conflicts of interest.

## Supporting information


**Data S1:** PRISMA 2020 checklist.

**Table S1:** The search strategy used in the different databases utilized.
**Table S2:** Theme 1. Empowerment and personal control.
**Table S3:** Theme 2. Consciousness and lifestyle changes.
**Table S4:** Theme 3. Professional‐patient interaction improvements.
**Table S5:** Theme 4. Personal user matters.
**Table S6:** Theme 5. Technology configuration.
**Table S7:** Theme 6. Economic and accessibility matters.
**Table S8:** Theme 7. Professional‐patient interaction.
**Table S9:** Them 8. Social support.
**Table S10:** Theme 9. Professional care and follow‐up.
**Table S11:** Theme 10. Technical challenges.
**Table S12:** Theme 11. Improvements in the data provided and its management.
**Table S13:** CERQual Assessment Table: Confidence in Qualitative Evidence Findings.

## Data Availability

The data that support the findings of this study are available from the corresponding author upon reasonable request.
