# Supplementary material for: Adults' Experiences and Perceptions of Electronic Devices for Diabetes Self‐Management: A Qualitative Evidence Synthesis
Source: Nurs Health Sci. 2026 May 4;28:e70348. doi: 10.1111/nhs.70348 (PMC13138877; doi:10.1111/nhs.70348)
Supplement: Supplementary file 1 — Data S1: PRISMA 2020 checklist. [file NHS-28-e70348-s002.pdf]

PRISMA 2020 Checklist

Section and Topic	Item #	Checklist item	Location where item is reported
**TITLE**			
Title	1	Identify the report as a systematic review.	Yes. The title identifies the report as a qualitative systematic review
**ABSTRACT**			
Abstract	2	See the PRISMA 2020 for Abstracts checklist.	The abstract includes objectives, methods, results, and conclusions
**INTRODUCTION**			
Rationale	3	Describe the rationale for the review in the context of existing knowledge.	Yes. The introduction provides a clear rationale for the review and its context within existing literature
Objectives	4	Provide an explicit statement of the objective(s) or question(s) the review addresses.	The objectives are explicitly stated in the introduction
**METHODS**			
Eligibility criteria	5	Specify the inclusion and exclusion criteria for the review and how studies were grouped for the syntheses.	Inclusion and exclusion criteria are clearly specified in the methods section
Information sources	6	Specify all databases, registers, websites, organisations, reference lists and other sources searched or consulted to identify studies. Specify the date when each source was last searched or consulted.	Databases searched are Medline, Scopus, Web of Science, CINHAL, ProQuest, and PsycINFO, with a search up to December 2024
Search strategy	7	Present the full search strategies for all databases, registers and websites, including any filters and limits used.	Yes. In supplemental table 1
Selection process	8	Specify the methods used to decide whether a study met the inclusion criteria of the review, including how many reviewers screened each record and each report retrieved, whether they worked independently, and if applicable, details of automation tools used in the process.	Selection was performed independently by two reviewers using COVIDENCE software, resolving

Section and Topic	Item #	Checklist item	Location where item is reported
			discrepancies with a third reviewer
Data collection process	9	Specify the methods used to collect data from reports, including how many reviewers collected data from each report, whether they worked independently, any processes for obtaining or confirming data from study investigators, and if applicable, details of automation tools used in the process.	Data were extracted using a structured spreadsheet, ensuring transparency
Data items	10a	List and define all outcomes for which data were sought. Specify whether all results that were compatible with each outcome domain in each study were sought (e.g. for all measures, time points, analyses), and if not, the methods used to decide which results to collect.	Data collected included study design, methodology, sample characteristics, and key themes
	10b	List and define all other variables for which data were sought (e.g. participant and intervention characteristics, funding sources). Describe any assumptions made about any missing or unclear information.	Data collected included study design, methodology, sample characteristics, and key themes
Study risk of bias assessment	11	Specify the methods used to assess risk of bias in the included studies, including details of the tool(s) used, how many reviewers assessed each study and whether they worked independently, and if applicable, details of automation tools used in the process.	JBI-QARI tool was used for quality assessment
Effect measures	12	Specify for each outcome the effect measure(s) (e.g. risk ratio, mean difference) used in the synthesis or presentation of results.	Not applicable as this is a qualitative synthesis
Synthesis methods	13a	Describe the processes used to decide which studies were eligible for each synthesis (e.g. tabulating the study intervention characteristics and comparing against the planned groups for each synthesis (item #5)).	A thematic synthesis approach was used following Thomas and Harden’s method.
	13b	Describe any methods required to prepare the data for presentation or synthesis, such as handling of missing summary statistics, or data conversions.	Not applicable
	13c	Describe any methods used to tabulate or visually display results of individual studies and syntheses.	The results are presented in structured tables summarizing study characteristics, methodologies, and key findings. A PRISMA flow diagram is included to illustrate the

Section and Topic	Item #	Checklist item	Location where item is reported
			study selection process.
	13d	Describe any methods used to synthesize results and provide a rationale for the choice(s). If meta-analysis was performed, describe the model(s), method(s) to identify the presence and extent of statistical heterogeneity, and software package(s) used.	Not applicable
	13e	Describe any methods used to explore possible causes of heterogeneity among study results (e.g. subgroup analysis, meta-regression).	Not applicable
	13f	Describe any sensitivity analyses conducted to assess robustness of the synthesized results.	JBI-QARI tool
Reporting bias assessment	14	Describe any methods used to assess risk of bias due to missing results in a synthesis (arising from reporting biases).	Not applicable
Certainty assessment	15	Describe any methods used to assess certainty (or confidence) in the body of evidence for an outcome.	Not applicable
**RESULTS**			
Study selection	16a	Describe the results of the search and selection process, from the number of records identified in the search to the number of studies included in the review, ideally using a flow diagram.	A PRISMA flow diagram presents the selection process
	16b	Cite studies that might appear to meet the inclusion criteria, but which were excluded, and explain why they were excluded.	Yes
Study characteristics	17	Cite each included study and present its characteristics.	Detailed in Tables 2 and 3, listing study country, method, sample, and recruitment
Risk of bias in studies	18	Present assessments of risk of bias for each included study.	JBI-QARI assessment results are presented in Table 1
Results of individual studies	19	For all outcomes, present, for each study: (a) summary statistics for each group (where appropriate) and (b) an effect estimate and its precision (e.g. confidence/credible interval), ideally using structured tables or plots.	Findings are summarized with key themes and subthemes
Results of syntheses	20a	For each synthesis, briefly summarise the characteristics and risk of bias among contributing studies.	Thematic synthesis results are structured into main themes, subthemes, and participant quotes
	20b	Present results of all statistical syntheses conducted. If meta-analysis was done, present for each the summary estimate and its precision (e.g. confidence/credible interval) and measures of statistical heterogeneity. If comparing groups, describe the direction of the effect.	Not applicable
	20c	Present results of all investigations of possible causes of heterogeneity among study results.	Not specifically discussed
	20d	Present results of all sensitivity analyses conducted to assess the robustness of the synthesized results.	Not specifically discussed

PRISMA 2020 Checklist

Section and Topic	Item #	Checklist item	Location where item is reported
Reporting biases	21	Present assessments of risk of bias due to missing results (arising from reporting biases) for each synthesis assessed.	Not specifically discussed
Certainty of evidence	22	Present assessments of certainty (or confidence) in the body of evidence for each outcome assessed.	Not explicitly addressed, but methodological rigor was followed
**DISCUSSION**			
Discussion	23a	Provide a general interpretation of the results in the context of other evidence.	Findings are compared with existing literature, and study limitations are acknowledged
	23b	Discuss any limitations of the evidence included in the review.	Findings are compared with existing literature, and study limitations are acknowledged
	23c	Discuss any limitations of the review processes used.	Findings are compared with existing literature, and study limitations are acknowledged
	23d	Discuss implications of the results for practice, policy, and future research.	Yes, in conclusions
**OTHER INFORMATION**			
Registration and protocol	24a	Provide registration information for the review, including register name and registration number, or state that the review was not registered.	Registered in PROSPERO (CRD42024572860)
	24b	Indicate where the review protocol can be accessed, or state that a protocol was not prepared.	Registered in PROSPERO (CRD42024572860)
	24c	Describe and explain any amendments to information provided at registration or in the protocol.	Registered in PROSPERO (CRD42024572860)
Support	25	Describe sources of financial or non-financial support for the review, and the role of the funders or sponsors in the review.	No specific funding received
Competing interests	26	Declare any competing interests of review authors.	No competing interests declared
Availability of data, code and	27	Report which of the following are publicly available and where they can be found: template data collection forms; data extracted from included studies; data used for all analyses; analytic code; any other materials used in the review.	Supplementary material and Data

PRISMA 2020 Checklist

Section and Topic	Item #	Checklist item	Location where item is reported
other materials			are available upon request from the authors

From: Page MJ, McKenzie JE, Bossuyt PM, Boutron I, Hoffmann TC, Mulrow CD, et al. The PRISMA 2020 statement: an updated guideline for reporting systematic reviews. BMJ 2021;372:n71. doi: 10.1136/bmj.n71. This work is licensed under CC BY 4.0. To view a copy of this license, visit https://creativecommons.org/licenses/by/4.0/
