# Supplementary material for: Adults' Experiences and Perceptions of Electronic Devices for Diabetes Self‐Management: A Qualitative Evidence Synthesis
Source: Nurs Health Sci. 2026 May 4;28:e70348. doi: 10.1111/nhs.70348 (PMC13138877; doi:10.1111/nhs.70348)
Supplement: Supplementary file 2 — Table S1: The search strategy used in the different databases utilized. Table S2: Theme 1. Empowerment and personal control. Table S3: Theme 2. Consciousness and lifestyle changes. Table S4: Theme 3. Professional‐patient interaction improvements. Table S5: Theme 4. Personal user matters. Table S6: Theme 5. Technology configuration. Table S7: Theme 6. Economic and accessibility matters. Table S8: Theme 7. Professional‐patient interaction. Table S9: Them 8. Social support. Table S10: Theme 9. Professional care and follow‐up. Table S11: Theme 10. Technical challenges. Table S12: Theme 11. Improvements in the data provided and its management. Table S13: CERQual Assessment Table: Confidence in Qualitative Evidence Findings. [file NHS-28-e70348-s001.docx]

**Supplemental tables**

**Supplemental Table 1**

The search strategy used in the different databases utilized.

| Database | Search strategy |
| --- | --- |
| Medline (PubMed)  Filter: Adult: 19+ years | ((Self-management[Title/Abstract]) AND (Diabetes[Title/Abstract] OR "diabetes mellitus"[Title/Abstract] OR "diabetes type 1"[Title/Abstract]) AND (app[Title/Abstract] OR device[Title/Abstract] OR eHealth[Title/Abstract] OR mHealth[Title/Abstract] OR mobile[Title/Abstract] OR phone[Title/Abstract] OR smartphone[Title/Abstract] OR application[Title/Abstract] OR smartwatch[Title/Abstract] OR technology[Title/Abstract] OR computer[Title/Abstract] OR internet[Title/Abstract]) AND ("qualitative study"[Title/Abstract] OR "qualitative research" [Title/Abstract] OR perception[Title/Abstract] OR "point of view"[Title/Abstract] OR sensation [Title/Abstract] OR experiences[Title/Abstract] OR Barriers[Title/Abstract] OR Facilitators[Title/Abstract])) NOT ("Diabetes Mellitus, Type 2" OR "TYPE 2 DIABETES" [Title/Abstract] OR “Gestational diabetes mellitus”[Title/Abstract] OR “Pregnant women” [Title/Abstract] or HOSPITALIZED [Title/Abstract] OR HOSPITALIZATION [Title/Abstract] OR DISABLED [Title/Abstract]) |
| Scopus | (TITLE-ABS-KEY(Self-management) AND TITLE-ABS-KEY(Diabetes OR "diabetes mellitus" OR "diabetes type 1") AND TITLE-ABS-KEY(app OR device OR eHealth OR mHealth OR mobile OR phone OR smartphone OR application OR smartwatch OR technology OR computer OR internet) AND TITLE-ABS-KEY("qualitative study" OR "qualitative research" OR perception OR "point of view" OR sensation OR experiences OR Barriers OR Facilitators) AND NOT TITLE-ABS-KEY("Diabetes Mellitus, Type 2" OR "TYPE 2 DIABETES" OR "Gestational diabetes mellitus" OR "Pregnant women" or HOSPITALIZED OR HOSPITALIZATION OR DISABLED)) AND ( LIMIT-TO ( EXACTKEYWORD,"Adult" ) ) |
| Web of Science | (Self-management AND (Diabetes OR “diabetes mellitus” OR “diabetes type 1”) AND (app OR device OR eHealth OR mHealth OR mobile OR phone OR smartphone OR application OR smartwatch OR technology OR computer OR internet) and (Barriers OR Facilitators OR “qualitative research” OR perception OR experiences OR “point of view” or sensation OR "qualitative study") ) (Topic) NOT ("Diabetes Mellitus Type 2" OR "TYPE 2 DIABETES" OR “Gestational diabetes ” OR “Pregnant women” or HOSPITALIZED OR HOSPITALIZATION OR DISABLED) (Topic) and Adult (Mesh Headings) |
| CINHAL  Limiters - Age Groups: All Adult Expanders - Apply equivalent subjects Search Modes - Boolean/Phrase Without specifying a specific field Unchecking 'Also search within the full text of the articles' | ( (Self-management) AND (Diabetes OR "diabetes mellitus" OR "diabetes type 1") AND (app OR device OR eHealth OR mHealth OR mobile OR phone OR smartphone OR application OR smartwatch OR technology OR computer OR internet) AND ("qualitative study" OR "qualitative research" OR perception OR "point of view" OR sensation OR experiences OR Barriers OR Facilitators) ) NOT ( ("Diabetes Mellitus, Type 2" OR "TYPE 2 DIABETES" OR "Gestational diabetes mellitus" OR "Pregnant women" or HOSPITALIZED OR HOSPITALIZATION OR DISABLED) ) |
| ProQuest | noft(adult AND "Self-management" AND (Diabetes OR "diabetes mellitus" OR "diabetes type 1") AND (app OR device OR eHealth OR mHealth OR mobile OR phone OR smartphone OR application OR smartwatch OR technology OR computer OR internet) AND ("qualitative study" OR "qualitative research" OR perception OR "point of view" OR sensation OR experiences OR Barriers OR Facilitators)) NOT noft("Diabetes Mellitus, Type 2" OR "TYPE 2 DIABETES" OR "Gestational diabetes mellitus" OR "Pregnant women" OR HOSPITALIZED OR HOSPITALIZATION OR DISABLED) |
| PsycINFO  Specify by SubjectAge: - adulthood (18 yrs & older) Expanders - Apply equivalent subjects Search Modes - Boolean/Phrase Without specifying a specific field Unchecking 'Also search within the full text of the articles | ( (Self-management) AND (Diabetes OR "diabetes mellitus" OR "diabetes type 1") AND (app OR device OR eHealth OR mHealth OR mobile OR phone OR smartphone OR application OR smartwatch OR technology OR computer OR internet) AND ("qualitative study" OR "qualitative research" OR perception OR "point of view" OR sensation OR experiences OR Barriers OR Facilitators) ) NOT ( ("Diabetes Mellitus, Type 2" OR "TYPE 2 DIABETES" OR "Gestational diabetes mellitus" OR "Pregnant women" or HOSPITALIZED OR HOSPITALIZATION OR DISABLED) ) |

**Supplemental Table 2**

Theme 1. Empowerment and personal control.

| Subtheme | Codes | Verbalizations |
| --- | --- | --- |
| Self-control and independence | Diabetes self-control | Most participants experienced more control over their diabetes while using CGM, in line with their expectations. I can only be positive and in two sentences: “I was in the middle of a diabetes-burnout, and I am now sure of myself and that is what it brought me. This study I think is called ‘IN CONTROL’, well that's it” (Vloemans et al., 2017) |
|  | Liberty | ‘‘I Just Want to Be Freer, I Guess’’ This theme focused primarily on the participants’ feelings toward their self-management routine, specifically feeling as though they wanted more freedom about wearing an insulin pump and the consistency with which they felt they had to manage their illness basis daily (Oser et al., 2019) |
|  | Less dependence on others | The protection offered by CGM helped individuals become less dependent on their spouses and family members; participants reported that their loved ones were less worried (e.g., regarding nocturnal hypoglycaemia) while using CGM and slept better at night, even with the alarms (Vloemans et al., 2017)  "I can manage my diabetes without relying so much on doctors." (Stawarz et al., 2023) |
|  | Facilitates recording data/glycaemic data | A participant with diabetes had used an online diabetes coach where they could input their blood glucose values to receive advice (…) mentioned that this could be helpful, especially for people who are just starting to use insulin injections, or when blood glucose values fluctuate a lot (Huygens et al., 2016) |
|  | Helps make decisions | A participant expanded on this sentiment by describing how the portal provided a critical step in their ability to take control of their health by facilitating access to their health results in a way they had not been able to before (…) "I don't like being in the dark about my healthcare, so I think the portal has helped me with that" (Marsh et al., 2020) |
| Decision-making and safety | Satisfaction with treatment | "I enjoyed using it," "It has offered me something that nothing else in my hustle and my avoidance has offered me". The whole group considered the app to be a useful addition to diabetes management, and some said they enjoyed using it (Knight et al., 2016) |
|  | Confidence | New technologies could have physical and psychological benefits in terms of better management of blood glucose (“My numbers have improved very significantly and if I look back at the sort of trace of my numbers sort of over the last four years it’s very clear that it directly coincides with you know a few weeks a few months after I started using [the FGM]”…), and greater feelings of confidence and control (“…in sort of psychological terms the confidence you can have is immeasurably better and it’s all because of the piece of the magic of being able to predict trends rather than just tell you what’s going on”) (Nettleton et al., 2022)  "With AI, I feel like I have constant support." (McFadden et al., 2024) |
|  | Continuous feedback | “Well, I just wanted to see where my blood sugar levels were and be able to track everything in one place, what I was eating, my activity level, my insulin doses, and then be able to see right away that it went down when you went and walked 2 miles, and compare that with a day where I’m sitting at my desk all day. It helped me understand how to better adjust my insulin doses, reflect better, or have more control and fewer fluctuations” (Ritholz et al., 2019) |
|  | More security and fewer worries | “That means I can do the things I like more when I want to. And that’s just a fantastic sensation”  "Continuous glucose monitoring devices and telemedicine systems provide reassurance and reduce patients' uncertainty” (Vitale et al., 2024) |
| Educational and communication aspects | Educational tool | Patients liked different types of educational materials. Some liked to see videos, while others liked reading articles (Zhang et al., 2018). Participants described Sugar Sleuth as an empowering educational tool which informed their lifestyle options and promoted their participation in diabetes self-care activities (Ritholz et al., 2019)  "Patients see the use of technology as a useful educational tool, but they mention that its effectiveness depends on their mood and consistency in usage." (Jensen et al., 2023) |
| Good glycaemic control | Analysing data when facing problems | “The first thing with a high or low is to figure out how this happened and why it happened . . . I look at the data.” (Martyn-Nemeth et al., 2019) |
|  | Depending on the mood | The ability to manage and cope with T1D was dependent on maintaining a positive emotional state. On days when participants struggled with the psychological demands of the condition, their ability to self-manage suffered: “It depends on how I’m feeling that day as to how well I manage to cope with my diabetes” (Nettleton et al., 2022) |
|  | Unpredictable | They also spoke about the unpredictability of their illness, specifically not knowing when their blood glucose level would drop too low. This caused participants to worry because many negative health consequences can occur from having a low blood glucose level. Each day for a diabetic is unpredictable. One could experience extremely low blood sugar without a clear explanation as to why it happened. Also, depending on the person, they may not recognize the symptoms of low blood sugar until it is too late, creating psychological impacts due to constant worries (Clausi & Schneider, 2017) |
|  | Mindfulness | “Having that level of mindfulness, staying in the present more, not beating myself up about what I’d maybe done in the past that hasn’t helped you know not worrying about what might happen in the future you know, will I lose my sight? Will I lose my toe? You know at what point will diabetes affect me going forward? Trying not to stay in that space but appreciate what is now you know it’s really important actually” (Nettleton et al., 2022) |
|  | Physical activity | Some physical concerns and inconveniences were mentioned, such as wearing the sensor and with certain clothes (or without), during exercise, sleep and while being intimate with their partner (Vloemans et al., 2017) |
|  | Diet | “…having a low-carb diet helped me face the situation, because having better blood sugar levels makes me feel better about (DT1), but what I’d say is that I’m struggling, I guess, to keep up with the diet. That can make you feel like you’re not facing it because when you see your blood sugar levels going up and down, you feel like you’re not taking control like you’re supposed to” (Nettleton et al., 2022)  "I can see that it's stable, but light activity might mean I need to eat soon." (McFadden et al., 2024) |

**Supplemental Table 3**

Theme 2. Consciousness and lifestyle changes.

| Subtheme | Code | Verbalizations |
| --- | --- | --- |
| Awareness and understanding of diabetes | Awareness of hormone and stress effects | Greater consciousness about the effects of hormones and stress, along with obtaining information about the effects of insulation and duration (Sorgard et al., 2019) |
|  | Consciousness of insulin effects and doses | "The use of sensors and AI systems helped improve awareness of insulin effects and dosing” (Vitale et al., 2024)  "Now I know how my body reacts to different insulin doses in various situations” (Persson et al., 2022) |
|  | Greater hypo/hyperglycaemia awareness | CGM helped improve glycaemic control via more consciousness and attention to blood glucose levels. Trend data made it clearer when blood glucose levels were outside the proper range. Participants described how CGM data let them stay on top of hyperglycaemia and hypoglycaemia (Sorgard et al., 2019) |
|  | Diabetes changing life perceptions | Participants also spoke about how type 1 diabetes having has made them realize that they should appreciate life and experience it to its fullest because you never know what could happen. ‘‘It just makes me, it just changed my perception on things, like on life in general, on kind of like the whole living life to its fullest type thing’’. Many also described feelings ‘‘lucky’’ that type 1 diabetes is manageable (Clausi & Schneider, 2017) |
| Daily improvements | Facilitating everyday life | “The challenge for me, before starting with the sensor, was that I didn’t take the time... to test; when it’s impractical for me during a busy work schedule, or a normal day or whatever, I didn’t prioritize it.” (…) The effects of exercise on blood glucose levels are a lot more visible. The trendy information was particularly useful. This lets participants take part in different activities and feel more secure and less concerned. They described depending less on planning and getting to be more spontaneous: … “and that means I can do the stuff I like when I want to. And that sensation’s simply fantastic.” (Sorgard et al., 2019)  "If I were going to a meeting at work, for example, and [it was predicted that my blood glucose would drop], I would probably have something sweet just to avoid the risk of having hypoglycaemia during the meeting.". (McFadden et al., 2024) |
|  | Glycaemic improvements | “In real life, I probably won’t get up every morning and look all night and say: ‘Oh, look, I’m up, I’m down...’ It’s just not happening. Maybe I don’t understand how to use all the data, but just that arrow going up and down and telling you what’s happening now is enough for you to do things” (Ritholz et al., 2019) |
|  | Psychological improvements | New technologies could have physical and psychological benefits in terms of better blood glucose management (“My numbers have improved very significantly and if I look back at the sort of trace of my numbers sort of over the last four years it’s very clear that it directly coincides with, you know, a few weeks, a few months after I started using” (Nettleton et al., 2022)  "Technologies such as sensors and insulin pumps have made patients' daily lives easier, enabling improvements in glycaemic control and emotional stability." (Jensen et al., 2023) |
|  | Routines | “You go to work, and you think your day is going to be a certain way; it never goes the way that you planned it. And so there’s these expectations: I’ll have time for that lunch or to test your blood sugar, look at your CGM every half hour. And you don’t because there’s always people and things going on.” [30]  "It might seem like it's not working, but it actually is, because if this happens while the app is getting to know you, it could be assimilating this information. And if you go through the same situation again later, it will have learned and might be able to suggest how many carbohydrates you should take before going to the gym to avoid hypoglycaemia. It's part of the learning process." (Stawarz et al., 2023) |
|  | Sensor and Pump improve sleep | "Participants noted that these technologies improve their sleep quality by reducing nighttime concerns’ (Markowitz et al., 2024)  “I think sleep for me, for many years, was a major fear. I think the thing that changed sleep the most, besides being married and having someone there with me, is CGM” (Martyn-Nemeth et al., 2019) |
|  | Freedom through technology | “(FGM) is as helpful to me on a day-to-day level as a wheelchair would be if I lost a leg. I mean it's hard to describe how useful it is as a technology and how valuable it is to me in terms of enabling me to live my life.” (Nettleton et al., 2022)  "Many mentioned that technology gives them a greater sense of freedom and autonomy in their daily routine." (James et al., 2023) |

**Supplemental Table 4**

Theme 3. Professional-patient interaction improvements.

| Subtheme | Code | Verbalization |
| --- | --- | --- |
| Communication | Sharing information | “I can download it, connect to the Wi-Fi, download it as a PDF document right away, and send it to my doctor within half an hour and it’s done. It’s easy to use.” (Franklin et al., 2019)  "I love the idea—though I use this term lightly—of an online library that holds all these resources our team has or that others have contributed. Because you can search for things on Google, but it's nice to feel like you're getting something from a slightly more legitimate source.". (Markowitz et al., 2024) |
|  | Improving communication | “It’s also been very useful when I’m seeing my doctor. Instead of just looking at a static list of blood sugar and insulin doses, I look at the graph and adjust. On my last visit, the doctor looked at the numbers first, but then he looked at the graph and just said: ‘I think you’re really doing a great job because I don’t see any wild fluctuations like we used to see. There are still some adjustments to make, but it’s great information and you’re doing a great job.’ And that’s the first time my doctor said I was doing a great job.... I think that all this is because we have access to more data points, more information” (Ritholz et al., 2019) |

**Supplemental Table 5**

Theme 4. Personal user matters.

| Subtheme | Codes | Verbalizations |
| --- | --- | --- |
| Consideration of technology | Depends upon the personal value attributed to the technology | “I should imagine someone who’s technical-minded then I think they could have a lot of fun, you know tracking patterns and trends and working out algorithms and things like that but for someone who is in their 50s or 60s who has just developed type 2 and then eventually is put on insulin and has to record glucose levels, I don’t know, I don’t know” (Waite et al., 2013)  "For some, AI and algorithms are fascinating, but for others, they can be a burden." (McFadden et al., 2024) |
| Mood | Depends upon mood | ‘‘I do my best to stay positive’’ Participants discussed the benefits and important lessons that they have learned from having to cope with their illness. Many participants spoke about reflecting on their experiences and realizing that they have come a long way with their management and that it was something that they were particularly proud of. “I would say to myself ‘No. You’re doing great. You’re able to manage all these new things that come with diabetes, and you are managing them very well within range of sugar levels.’ And I found it would pick me up into a much better mood” (Clausi & Schneider, 2017) |

**Supplemental Table 6**

Theme 5. Technology configuration.

| Subtheme | Code | Verbalization |
| --- | --- | --- |
| Unifying devices | Recording on smartphone | The group agreed that storing the settings and blood glucose diary data on the web would be a more effective way to manage their diabetes diary (…). Some participants referred to the web-based storage of other phone data and mentioned that web-based storage of RapidCalc settings on the phone would also be useful to ensure data security in case of device loss. (Knight et al., 2016) |
| Device manageability | Ease of use | Young people and children appeared to more intuitively explore and appraise the methods of data entry and features, such as the opportunity to be able to visualize personal trends within a graph. “Very good app, I think by the end of it I’d changed my mind about how much I liked it (…) It was a very useful way of being able to look at your blood sugars on the graph, seeing, you know, looking for patterns, watching out for things that could happen again” (Waite et al., 2013) |
|  | Sensor data management | Most participants affirmed that they used the time point data which was available on the screen and perceived the ROC arrows and trend graphics as the most valuable parts of CGM. None of them used the retrospective visualization via downloading MCG data; some said that they felt more comfortable reviewing the retrospective analysis together with their doctor or nurse in the diabetes clinic (Sorgard et al., 2019) |
|  | Need to be intuitive | Intuition– some of the older adults in the study related differently to their experience of using the app, compared with the children and young people. “… But for me, it was a machine that I had to sort of do a favour to you to put the results in, which is fine, I didn’t mind doing but it’s not something I would do for myself…” (Waite et al., 2013) |
|  | Need to be quick and easy to use | “Since I don’t have time, I just want something quick and easy to use. Just something quick and easy to use… and then I can analyse it later. I think that, but for me, because I’m so busy all the time if it’s something quick and easy, I’d do it.” (Franklin et al., 2019) |
| Design and strategies | More value in experiential learning (experience-based) than the doctor | It is interesting also to think about the kinds of information that people are seeking. In a study of online health-information seekers, Kivits19 found that there was a very ‘everyday dimension’ to the information people were seeking, and that ‘experiential knowledge’ was valued, sometimes above medical expertise: “I’m not saying the doctors are no use and I wouldn’t ask them anything, but there are certain types of question I would ask them and other types of question where I would go to other users first and I know where I’d be most likely to find the answer” (Armstrong & Powell, 2009) |
|  | Use gamification | “Besides the fact that you know, children and young people like to play games and use computers and iPods and things, you know, these are things that they use all the time, it might encourage them a bit more to use it” (Markowitz et al., 2024) |
|  | Being user-friendly | “I think it would be great if it could be even more user-friendly. For example, I must go into the portal to see if something is updated, I’d like it if there is a notification that I get when said test result comes in/uploaded (so I don’t have to check in as frequently when waiting for results). It would be nice if you saw a provider that wasn’t your usual provider like urgent care that information would be documented on my portal. Might be complicated to manage but that would be ideal if possible” (Marsh et al., 2020) |
| Managing information | Receiving positive and encouraging feedback from the app | “To pull forward and give you some feedback so that, because the other thing is you don’t get any feedback until your next visit and if your visit is in 12 months then the incentive to complete your record book, I think, is probably more difficult” (Waite et al., 2013) |
|  | Easier glucose level data access | CGM offered easy access to information about blood glucose levels, saving time and energy. Patients did not have to interrupt other activities or plans, as is necessary with a finger puncture test (Sorgard et al., 2019) |
|  | Data visualization ability | Being able to visualize the relation between entries, including GS, insulin, exercise, and food, was a main characteristic to facilitate making decisions (Franklin et al., 2019) |

**Supplemental Table 7**

Theme 6. Economic and accessibility matters.

| Subtheme | Codes | Verbalizations |
| --- | --- | --- |
| Economic matters | Free access to materials | “[The photograph] just reminded me of how grateful I am for the NHS in general like the fact that I get all this stuff for free, this hefty bag of goodies that help me live and you don’t have to pay a penny for it.” (Nettleton et al., 2022)  "My pump recently went out of warranty, so I had to go through the process of getting a new one, and I think the entire cost of the pump goes toward my deductible, but then there's also a copay… no, no, no, it's not a copay because a copay would be a fixed amount” (Vitale et al., 2024)  "The first thing is that these apps should be free for patients, especially for older adults” (Dehnavi et al., 2022) |
|  | Universal access | “What I used to hate (…) is doing the blood test, because it was like waiting for test results every time you did it and whether or not you were doing well or not doing well you were sort of judged by that test result and it was very much a moment of isolation a blood test result whereas at least with this (FGM) what you can see is a pattern, so you can see if it’s going up or coming down, you can make better judgements about how you then manage that and I think it’s massively sad that it’s not available to everyone but who knows, hopefully, it will in the future.” (Nettleton et al., 2022) |

**Supplemental Table 8**

Theme 7. Professional-patient interaction.

| Subtheme | Codes | Verbalizations |
| --- | --- | --- |
| Support | Support from health professionals | “I am happy to receive information from my doctor, or my mom. I am not ready to take on all that is needed to manage my health. Diabetes is a big deal. I need all the help I can get. Most people want to be on their own with their life at this age (22 years) but I have to say diabetes changes everything. If I didn’t have diabetes, I think I would be a lot more independent but quite frankly, I am worried about not waking up at night due to a low blood sugar and I need my mom to help me manage my diabetes.” (Marsh et al., 2020)  "I think this prepares both you and the doctor and nurse because they also see it before the appointment. This way, conversations can be more focused instead of talking about the same things every time." (Jensen et al., 2023) |
| Making decisions | Professionals’ influence | “I had a really good diabetes specialist nurse (DSN) to start with (…) then I was kind of put in this limbo and […] I would feel quite negative, I would leave and even if my A1c was down there’d be something else that was an issue (…) I would always leave feeling very despondent actually and feeling like I’d been told off, so this day when I (…) finally ended up on the pump system was huge because all of a sudden I was seeing the top two [consultants] (…) it was almost like being in a different hospital, they would listen to me, they would understand my point of view and we would discuss stuff and that was far better than being made to feel stupid (…)”(Nettleton et al., 2022) |

**Supplemental table 9**

Them 8. Social support.

| Subtheme | Code | Verbalizations |
| --- | --- | --- |
| Online support | Online support | Online peer support helps drive adults with Diabetes Type 1 to exercise  "And it's much faster to get answers on social media rather than scheduling appointments with specialists... Like all these little issues we deal with daily that don't appear in textbooks and that healthcare professionals don't necessarily relate to. So, it's nice to get different perspectives from different people."  (Markowitz et al., 2024) |
| Peer support | Peer support | They thought that peer support could help them trade glycaemic control strategies and emotional experiences. Some even thought that patient experiences were more important than consulting doctors, because patients’ experiences were specific to each person and practice. (Zhang et al., 2018) |
| Support from family and friends | Family and friends | “I think people might be more drawn to using these kind of things if they don't have somebody like I did with Mum and Dad, who sort of really cared for the whole thing, got as much information as they could (…) other people might have different family backgrounds, (…) they might have to look for it themselves, and that's going to be difficult for them, but I mean having read the guys' [comments] and people they can talk to [the online community] who know what they're talking about, [it is] quite comforting for them to have so they probably use that facility quite a lot, eh I know I would if I didn't have the support of Mum and Dad” (Fergie et al., 2016) |

**Supplemental table 10**

Theme 9. Professional care and follow-up.

| Subtheme | Code | Verbalizations |
| --- | --- | --- |
| Care continuity | Not all professionals are included | The critique that the portal only presents information related to their T1DM may indicate several different situations for the participants. One is that the participants are seeing an endocrinologist who is not part of their primary care system, so their portal only has specialty information (Marsh et al., 2020) |
|  | Need for mental health support | “I didn't look it up [online], (…) after going to see my GP (general practitioner) and then (mental health professional), I didn't look at any other ways because [she] helped a lot (…) whereas if it was getting worse and [she] wasn't helping, I would have (…). Whereas if it [had not] helped, then I'd still be looking for that, for that open door to kind of make me feel better” (Fergie et al., 2016) |
|  | Lack of psychological support modules | “Another thing is the psychological, a psychological module for patients. I have lots of apps on my mobile phone. Almost all of them are about knowledge, how to control my blood sugar. For child mental health care, there’s no psychological module” (Zhang et al., 2018) |
|  | Distrust of unknown professionals | However, most users did not ask HCPs via the application due to not trusting unknown doctors. Communicating with the app via writing words was inconvenient, with low communication efficiency. Consultations had to be paid for, feedback was not timely, and consultation impact was low (Zhang et al., 2018) |
| Professionals’ training | Professionals did not know how to manage the apps | Another participant commented that her health-care team could do more to bridge the gap between offering tools to patients and educating patients on how to use them to access specific information: “There needs to be more information about the portal and how to transition from child to adult healthcare. Maybe a sign at the NP office or in the health centre at college.” (Marsh et al., 2020) |
| Professionals’ motivations | Lack of feedback from professionals such as a problem | “I’ve received negative opinions and comments from (health professionals) who haven’t bothered understanding why I haven’t controlled my diabetes. They’ve just judged me and made me feel like I’m failing with my diabetes” (Ng et al., 2017) |

**Supplemental Table 11**

Theme 10. Technical challenges.

| Subthemes | Codes | Verbalizations |
| --- | --- | --- |
| Device management and configuration | Problems with handling | “I used to note it from the machine, but then the book gets lost, there aren’t enough pages, you can’t extend it or put in more, you can’t add a note, you have to write it really small, it’s all very disorganized and takes lots of time.”(Franklin et al., 2019) |
|  | Errors in data recording and synchronization | “…it’s just not reading right and it’s making me make bad decisions (surrounding insulin dosing) which is the opposite of what it’s supposed to do”. This caused frustration when needing to revert to finger prick blood glucose monitors, prohibiting the freedom that modern technology provided. “One thing about the (FGM) that hasn’t really improved my coping is that it does make me want to check (blood glucose levels) more so I’ve gone from testing me like 8 or 10 times a day to sometimes 20 or 30 times a day because I have that option and it makes me really hypervigilant about my blood sugars (…) so it makes me think about diabetes a lot more I think” (Nettleton et al., 2022) |
|  | Restrictions in glycaemic ranges | Frustration was expressed at various apps’ inability to meet personal requirements; for example, overly restrictive limits on ranges of numbers, such as units of insulin and for blood glucose levels. “You couldn’t input high figures so the maximum number of millimoles that it would register would be 25. Now sometimes my readings are way above 25…” (Waite et al., 2013) |
|  | Concern for repairs and costs | Excessive use of sensors due to failures and dislodging generated concerns about additional costs and reimbursement fixes (Sorgard et al., 2019) |
| Access and funding | Having to pay for some functions | “I once tried to make a doctor’s appointment in the Weltang app. But during those few minutes, it needed to load, so I went out. An unknown doctor consults it, but must pay” (Zhang et al., 2018) |
|  | Patients do not have all the resources (apps) they need | These data suggest that all providers should take a more active role in educating patients on all tools and technology available to patients with chronic diseases. In addition, these data may indicate that providers need to receive training throughout their course of practice to ensure they are adequately skilled in advising patients on how to manage their health outside the office of the provider (Marsh et al., 2020) |
|  | Universal access | “What I used to hate (…) is doing the blood test, because it was like waiting for test results every time you did it and whether or not you were doing well or not doing well you were sort of judged by that test result and actually it was very much a moment of isolation a blood test result whereas at least with this (FGM) what you can see is a pattern, so you can see if it’s going up or coming down, you can make better judgements about how you then manage that and I definitely think it’s massively sad that it’s not available to everyone but who knows, hopefully it will in the future.” (Nettleton et al., 2022) |
| Device size and form | Device visibility | “I hate technology. I’m an outdoorsy nature kind of person. But technology really has been my saving grace. I had to embrace the pump. I had to embrace the CGM. I had to question really smart people to learn from them. Even though I thought I knew my diabetes the best. But I didn’t.” (Martyn-Nemeth et al., 2019) |
|  | Carrying the technology as a burden | Using and transporting supplies and tech for diabetes is a burden, which can be reduced if the family helps. (Oser et al., 2019) |
|  | Body image concerns /Managing body image problems | Feeling that self-management should be hidden from others was isolating: “From the outside no-one sees you like no-one realizes what we go through as TIDM, and all the trouble caused like it is largely a hidden condition (…) (the photograph is) revealing the hidden stuff that you don’t see. I’m quite self-conscious about my injection scars” (Nettleton et al., 2022) |
|  | Stigmatizing configuration | Joe also used Facebook to contribute health-related content, acknowledging the potential undesirability of using the site in this way: “You know, I will quite often just sort of tell Facebook that I'm feeling anxious or I'm feeling down, or I'm just confused and things like that. I try to avoid what they call ‘vague booking’, which is, you know, posting something vague sounding so that people will ask you a question about it” (Fergie et al., 2016) |
|  | Intimate relations | Some physical concerns and inconveniences were mentioned, such as wearing the sensor and with certain clothes (or without), during exercise, sleep and while being intimate with their partner (Vloemans et al., 2017) |
|  | Sensor-related management problems | Discomfort and pain when inserting the sensor made some people quit using it: “When you’ve got something that should help you and ought to work, but you wind up with lots of problems, it becomes so negative that you can’t bear to keep using it (CGM), and I just put it away” (Sorgard et al., 2019) |
|  | Discomfort | Some physical concerns and inconveniences were mentioned, such as wearing the sensor and with certain clothes (or without), during exercise, sleep and while being intimate with their partner (Vloemans et al., 2017) |
| Alarms | Embarrassment when responding to alarms in public | Some participants mentioned feeling annoyed or embarrassed when having to respond to the CGM in the presence of others (Vloemans et al., 2017) |
|  | Alarms disrupting sleep | The sensor or pump was another source of sleep disruption for some participants, with 1 describing flashing lights from an expired sensor and several describing high or low glucose alarms. One participant noted: “I had to change my sensor last night. It expired very late at night. So, I had to change it, and it was flashing, so I put it under a towel so I wouldn’t have to keep seeing it” (Griggs et al., 2020) |
|  | False alarms | While useful strategies to cope with the condition, reminders were also not infallible. Missing alarms could cause mistakes and feelings of guilt (Nettleton et al., 2022) |
| Device information | Continually thinking about diabetes | “One thing about the (FGM) that hasn’t really improved my coping is that it does make me want to check (blood glucose levels) more so I’ve gone from testing me like 8 or 10 times a day to sometimes 20 or 30 times a day because I have that option and it makes me really hypervigilant about my blood sugars (…) so it makes me think about diabetes a lot more I think” (Nettleton et al., 2022) |
|  | Frustration with seeing all data (film of reality instead of snapshots) | For some, CGM negatively affected their well-being and sense of control, because of being confronted with daily and between-day glucose fluctuations that they had previously not been aware of. “Fluctuations you previously were not aware of, you do notice with the sensor. And that is frustrating (…) you keep thinking why does it rise” (Vloemans et al., 2017) |
|  | Data recording as a burden | “If you’re manually recording while you’re out there, measuring blood sugar is inconvenient, but you’ve got to do it ... you’ll think it doesn’t matter. It’s just in the glucose measurer. It’s really heavy. But it can be transmitted automatically to the app, it’s convenient” (Zhang et al., 2018) |
|  | Data overload causes stress | It was mentioned by a few that they had become ‘somewhat obsessed’ by continuously checking the CGM, which added to ‘feeling like a diabetic’ instead of ‘knowing you have diabetes’ (Vloemans et al., 2017). The data available through downloading and visualizing retrospective analysis was also stressful for some. When seeing the numbers on paper, they felt like their mistakes and shortcomings were being documented (Sorgard et al., 2019) |
| Device reliability | Technical issues | A few participants did not experience an increased sense of control, mainly because they had difficulty trusting the device having experienced a technical failure (Vloemans et al., 2017) |
| Privacy | Security | What features would you like to see in the future development of mobile apps to support diabetes care? The features that participants would like to see in the future development of mobile apps could be categorised under the following themes – safety-netting and feedback (Waite et al., 2013) |
|  | Platform privacy | “Facebook, links into your sort of general, your wider, you know, Facebook profile, and it's (mental health issue) not something you would necessarily want to be public. And if someone's stalking you on Facebook, not stalking, but, you know, someone is looking at your Facebook page and they see that, that's not necessarily something you'll want them to see” (Fergie et al., 2016) |
| Fungible device material | Stayin on top of the fungible material | Many participants also described their self-management as an entity from which they could not escape and at times feeling ‘‘trapped’’ by their self-management. “I think it’s annoying that I must go upstairs to my room and get my supplies or bring it with me everywhere I go. I’m always carrying insulin and needles, so that I’m prepared. And then I see everyone else who just goes about their normal life, which I was used to for so long, just grab something and eat it without question. I think that is something so simple in people’s lives that I miss sometimes” (Clausi & Schneider, 2017)  "Honestly, I'm reordering and... juggling supplies... getting my Dexcom sensors... trying to order supplies for my pump and... insulin... and I have to get them from different places." (Vitale et al., 2024) |
| Device calibration | Calibration delays | “And then it’s calibrating and calibrating. After three hours, another calibration. In the end, I used it a long time, but there weren’t any readings, just calibration. And it’s very annoying, and I think it’s a lot better to just control my blood sugar level with a regular fingertip prick” (Sorgard et al., 2019) |
| Device insertion | Skin reactions | Some commented on skin irritation and problems attaching the sensor to the skin (Vloemans et al., 2017) |
|  | Sensor insertion pain | There were complaints about the need for calibration of the CGM system with SMBG and the unpleasant or painful insertion of the sensor (Vloemans et al., 2017) |
| Personalization | Not adaptable to all populations | Concerns were voiced that it could be too hard for poorer disabled people or those from different cultural environments to adopt this technology, since they lacked access to the same information or resources than people in the main technology (Ritholz et al., 2019) |
|  | Obsolete/ out of style | Many participants described the device as obsolete and passé, and felt that they had limited abilities to control the alarm functions (Sorgard et al., 2019) |
|  | Fragmented, non-holistic vision | When asked about how they currently use the patient portal or factors that would lead to increased portal usage, 20 participants indicated that they are looking for a platform that presents a more comprehensive picture of their health in concert with having the strategies to help them manage their care, rather than only being able to receive results from one doctor or only results about diabetes. These data suggest that patients want their health to be viewed holistically and not segmented based on provider, diagnosis, or illness (Marsh et al., 2020) |

**Supplemental Table 12**

Theme 11. Improvements in the data provided and its management.

| Subtheme | Codes | Verbalizations |
| --- | --- | --- |
| Information online | Including strategies to help manage DM | “I think I have the tools and apps I need. I use the Dexcom app, which is not through the provider but helps me manage and view how I’m doing with my diabetic health. An “all in one app” where my doctor could view my Dexcom app may be helpful but if I want my provider to view my numbers from my Dexcom I can give him a code that he can go to the Dexcom website and view my numbers for the past month or two months. I have never used this when emailing my provider but once a provider pulled up my numbers online while I was in the visit so she could check how I had been doing. If I have a problem and they could view it automatically that may be helpful.” (Marsh et al., 2020) |
|  | Seeking out diabetes information online | “If I had to find out something that I couldn't get an answer for from the public hospital system, I would look it up on Google. I had to Google to find out what would happen to my blood sugar levels at high altitudes because I was going to travel abroad” (Ng et al., 2017) |
|  | Needing to be able to filter Internet data | “What if you get people though who are giving you wrong advice? I mean… you know… you could get somebody coming in and saying ‘Well, I only increase my insulin by one unit when I have a cold’ you know? Or… but I mean you’ve got to be able to select and think ‘Well, I don’t think that’s quite right.” (Armstrong & Powell, 2009)  "I don't know how scientific this information is. They could give me false information, and I might use it—that's really bad. It could happen to me since I use these channels frequently.". (Dehnavi et al., 2022) |
| Visualizing personal data | Ability to visualize information | The ability to visualize the relation between entries, like GS, insulin, exercise, and diet, was a major trait enabling decision-making (Franklin et al., 2019) |
|  | Desire to access health data via the app | Patients expected to be able to access their electronic medical records (EMR) from the hospital through the app (e.g., to see their test results and their diagnosis and treatment records, and to schedule walk-in visits). This would be convenient, letting them build health records in the app and motivate them to continue using the app (Zhang et al., 2018) |
|  | Data recording as a burden | Although they thought that a mobile diabetes diary more convenient than a paper diary, most thought the manual entry was a chore. Patients wanted glucose data to be automatically transmitted to the apps (Zhang et al., 2018) |
| Contents | More comprehensible DM information | “I tried the portal once and frankly; it was too confusing. I must tell you I’m a big techy guy so if the patient portal is too confusing for me then it is too confusing for most people.” (Marsh et al., 2020) |
|  | Include notifications | “I think it would be great if it could be even more user friendly. For example, I have to go in to the portal to see something is updated, I’d like it if there is a notification that I get when said test result comes in/uploaded (so I don’t have to check in as frequently when waiting for result). It would nice if you saw provider that wasn’t your usual provider like urgent care that that information would be documented on my portal. Might be complicated to manage but that would be ideal if possible” (Marsh et al., 2020) |
|  | Wanting more individualized information | Reported needs for (online) communication were that communications should be direct, comprehensible, adapted to the patient, and done by a human being, not by a pre-programmed app (Huygens et al., 2016) |
| Unreliability | Reliable information | “There’s so much (info online). It’s hard to tell what’ll be useful and what won’t, and there’s also lots of people who make money with it. So it’d be good to know which programs and measurers are useful, that sort of thing” (Ng et al., 2017) |
|  | Need for evidence-based information | Participants valued evidence-based updates about various diabetes control aspects, like nutrition and exercise, as they moved towards positive health behaviour changes in response to maturing health perceptions (Ng et al., 2017) |
|  | Data provided by the app was not professional | Some patients thought that knowledge about diabetes in the apps was neither systematic nor professional. Patients did not know whether diabetes knowledge was exact. “It’s too varied. You can’t tell which one’s right. Since most of us get information online, I think precision’s more important for information on diseases” (Zhang et al., 2018) |

**Supplemental Table 13.**

CERQual Assessment Table: Confidence in Qualitative Evidence Findings

| Summary of review finding | Studies contributing to the review finding | Methodology | Coherence | Data adequacy | Relevance | Country | CERQual assessment of confidence in the evidence | Explanation of CERQual assessment |
| --- | --- | --- | --- | --- | --- | --- | --- | --- |
| Finding 1: Digital technology provides empowerment, autonomy, and personal control in the management of type 1 diabetes | (Armstrong & Powell, 2009; Clausi & Schneider, 2017; Fergie et al., 2016; Franklin et al., 2019; Huygens et al., 2016; Markowitz et al., 2024; Marsh et al., 2020; Martyn-Nemeth et al., 2019; Nettleton et al., 2022; Ng et al., 2017; Oser et al., 2019; Ritholz et al., 2019; Sorgard et al., 2019; Vloemans et al., 2017; Waite et al., 2013; Zhang et al., 2018) (Dehnavi et al., 2022; James et al., 2023; Jensen et al., 2023; Stawarz et al., 2023; Xie et al., 2023) (Barth et al., 2024; McFadden et al., 2024; Persson et al., 2022; Vitale et al., 2024) | Minor concerns | High | Sufficient | High | USA, Norway, China, Australia, UK Netherlands, Canada, Denmark, Iran, Sweden, Switzerland | High Confidence | This finding is supported by a large number of studies (more than 20), conducted in diverse geographical and cultural contexts, which provides high relevance and adequacy. The consistency across studies is high, with no notable contradictions, which supports coherence. Additionally, the studies demonstrate good methodological quality. |
| Finding 2: The use of ICT facilitates positive lifestyle changes and reduces emotional burden | (Barth et al., 2024; Clausi & Schneider, 2017; Dehnavi et al., 2022; Franklin et al., 2019; Griggs et al., 2020; Huygens et al., 2016; James et al., 2023; Jensen et al., 2023; Knight et al., 2016; Markowitz et al., 2024; Martyn-Nemeth et al., 2019; McFadden et al., 2024; Nettleton et al., 2022; Ng et al., 2017; Oser et al., 2019; Persson et al., 2022; Ritholz et al., 2019; Sorgard et al., 2019; Stawarz et al., 2023; Vitale et al., 2024; Vloemans et al., 2017; Xie et al., 2023; Zhang et al., 2018) | Minor concerns | High | Sufficient | High | USA; Norway, UK, Netherlands, Australia, China, Canada, Netherlands, Denmark, Iran, Switzerland | High Confidence | This finding is also well supported by numerous high-quality studies, with consistent results showing improvements in health awareness, habits, and emotional well-being. The studies come from multiple regions, which enhances their transferability (relevance and adequacy) and strengthens coherence. No significant methodological limitations were identified. |
| Finding 3: Interaction with healthcare professionals improves through digital tools but depends on the level of support received. | (Franklin et al., 2019; Huygens et al., 2016; Jensen et al., 2023; Knight et al., 2016; Markowitz et al., 2024; Marsh et al., 2020; Nettleton et al., 2022; Ng et al., 2017; Ritholz et al., 2019; Sorgard et al., 2019; Vitale et al., 2024; Waite et al., 2013; Zhang et al., 2018) | Minor Concerns | Moderate | Moderate | High | Norway, USA, UK, Netherlands, Australia, China, Canada, Denmark | Moderate Confidence | Although a clear trend toward improved professional–patient relationships is observed, the results largely depend on the institutional context and the level of professional engagement. This introduces some variability (moderate coherence). Despite the overall good quality of studies, not all explore this aspect in depth. Relevance is good but not consistent across all contexts. |
| Finding 4: The adoption of ICT is influenced by personal factors, age, emotional state, and perceived value. | (Barth et al., 2024; Clausi & Schneider, 2017; Dehnavi et al., 2022; James et al., 2023; Jensen et al., 2023; Markowitz et al., 2024; Martyn-Nemeth et al., 2019; McFadden et al., 2024; Nettleton et al., 2022; Persson et al., 2022; Ritholz et al., 2019; Sorgard et al., 2019; Stawarz et al., 2023; Vitale et al., 2024; Vloemans et al., 2017; Waite et al., 2013; Xie et al., 2023) | Some concerns | Moderate | Moderate | High | Norway, Netherlands, Canada, USA, UK, Denmark, Sweden, Switzerland | Moderate Confidence | This finding is supported by methodologically sound studies, but there is clear variability in user experiences depending on age, technological skills, and emotional state, which affects coherence. Additionally, some studies do not address all factors comprehensively. Nevertheless, its relevance is high across different contexts. |
| Finding 5: Economic, technical, and accessibility barriers hinder the equitable implementation of ICT. | (Barth et al., 2024; Dehnavi et al., 2022; Sorgard et al., 2019; Vitale et al., 2024) | Some concerns | Low | Limited | Moderate | Iran, Switzerland, USA, Norway | Low Confidence | Few studies explicitly address this issue, and most come from specific contexts (Norway, Iran, USA, Switzerland), which limits their adequacy and global relevance. Additionally, coherence is limited, as the reported barriers vary significantly between countries. Although the evidence is valid, it is scarce and does not always explore the actual impact in depth. |
| Finding 6: Gaps in professional follow-up and psychological support negatively affect the patient experience. | (Barth et al., 2024; Dehnavi et al., 2022; James et al., 2023; Jensen et al., 2023; Markowitz et al., 2024; Marsh et al., 2020; Nettleton et al., 2022; Ng et al., 2017; Vitale et al., 2024; Xie et al., 2023; Zhang et al., 2018) | Some concerns | Moderate | Moderate | High | Canada, Denmark, Iran, UK, Switzerland, USA, China | Moderate Confidence | There is moderate support from several studies regarding the lack of continuity in professional support, although the degree of impact varies depending on the healthcare system. This introduces inconsistency (moderate coherence). Relevance is good, as it spans different contexts, but some studies address this issue only indirectly. |
| Finding 7: Technical and usability issues lead to frustration and mistrust in the continued use of ICT. | (Armstrong & Powell, 2009; Clausi & Schneider, 2017; Dehnavi et al., 2022; Fergie et al., 2016; Griggs et al., 2020; James et al., 2023; Jensen et al., 2023; Knight et al., 2016; Markowitz et al., 2024; Martyn-Nemeth et al., 2019; McFadden et al., 2024; Nettleton et al., 2022; Ng et al., 2017; Oser et al., 2019; Persson et al., 2022; Ritholz et al., 2019; Sorgard et al., 2019; Stawarz et al., 2023; Vitale et al., 2024; Vloemans et al., 2017; Waite et al., 2013; Xie et al., 2023; Zhang et al., 2018) | Minor concerns | High | Moderate | High | Canada, Denmark, Iran, UK, Sweden, USA, Norway, Australia, Netherlands | High Confidence | This finding is widely supported by a large number of studies conducted in various contexts. Negative experiences related to technical aspects (failures, complex interfaces, poorly managed data) are consistent and recurrent. Adequacy is strong, as the studies include participants of different ages and levels of digital literacy. Methodological quality is generally good. |
| Finding 8: Technology can facilitate social support and shared learning among peers. | (Armstrong & Powell, 2009; Clausi & Schneider, 2017; Dehnavi et al., 2022; Fergie et al., 2016; James et al., 2023; Markowitz et al., 2024; Marsh et al., 2020; Nettleton et al., 2022; Ng et al., 2017; Oser et al., 2019; Vitale et al., 2024; Xie et al., 2023; Zhang et al., 2018) | Minor concerns | Moderate | Moderate | High | UK, Canada, Iran, USA, China, Australia | Moderate Confidence | Studies consistently show that ICT facilitates sharing among people with type 1 diabetes, but they are fewer in number, and not all explore this aspect in depth. Coherence is good but limited by the amount of available data. Relevance is high, although some cultural settings may influence the applicability of the finding. |
